# Large role of anthropogenic climate change in driving smoke concentrations across the western United States from 1992 to 2020

**DOI:** 10.1073/pnas.2421903122

**Published:** 2025-12-02

**Authors:** Xu Feng, Loretta J. Mickley, Jed O. Kaplan, Makoto Kelp, Yang Li, Tianjia Liu

**Affiliations:** ^a^John A. Paulson School of Engineering and Applied Sciences, Harvard University, Cambridge, MA 02138; ^b^Department of Earth, Energy, and Environment, University of Calgary, Calgary, AB T2N 1N4, Canada; ^c^Department of Earth System Science, Doerr School of Sustainability, Stanford University, Stanford, CA 94305; ^d^Department of Environmental Science, Baylor University, Waco, TX 76798; ^e^Department of Geography, University of British Columbia, Vancouver, BC V6T 1Z2, Canada

**Keywords:** wildfires, anthropogenic climate change, smoke concentrations

## Abstract

Wildfire activity has increased dramatically in the western United States over the last three decades, having a significant impact on air quality and human health. However, quantifying the drivers of trends in wildfires and subsequent smoke concentrations is challenging, as both natural variability (NV) and anthropogenic climate change (ACC) play important roles. Here, we devise an approach involving observed meteorology and vegetation and a range of models to determine the relative roles of ACC and NV in driving burned area across the western United States. We also examine the influence of ACC on smoke concentrations. We estimate that ACC accounts for 33 to 82% of observed total burned area, depending on the ecoregion, yielding 65% of total fire emissions on average across the western United States from 1992 to 2020. In all ecoregions except Mediterranean California, ACC contributes to a greater percentage of burned area in lightning-ignited wildfires than in human-ignited wildfires. On average, ACC contributes 49% to smoke PM_2.5_ concentrations in the western United States from 1997 to 2020, and explains 58% of the increasing trend in smoke PM_2.5_ from 2010 to 2020. Northern California and areas in Oregon, Washington, and Idaho experience the greatest smoke concentrations attributable to ACC, averaging 40 to 66% of total PM_2.5_ over 2010–2020. Our work highlights the significant role of ACC in degrading air quality in the western United States and identifies those regions most vulnerable to wildfire smoke and thus adverse health impacts.

## Significance

Wildfire activity has increased dramatically in the western United States over the last three decades, leading to a significant impact on air quality and human health. This study highlights the substantial role of anthropogenic climate change (ACC) in driving these wildfires and the subsequent smoke concentrations in the western United States. Our results suggest that ACC contributes 33 to 82% to observed burned area in the western United States from 1992 to 2020. Such climate change also accounts for nearly half of surface smoke PM_2.5_ concentrations from 1997 to 2020 and explains 58% of the increasing smoke trend from 2010 to 2020. Our study calls attention to the threat that wildfires have on smoke concentrations and human health under a changing climate regime.

Wildfire activity has increased dramatically across the western United States during recent decades (1–3). The increasing magnitude and interannual variability of wildfires has been traced to a combination of anthropogenic climate change (ACC), natural variability (NV), and human-caused ignitions (4–7). As a result, the burden of wildfire smoke–a complex mixture of carbonaceous aerosols, other types of fine particulate matter (PM_2.5_), and trace gases–has also increased, eroding multidecadal efforts to reduce PM_2.5_ concentrations in most western states (8, 9). Although PM_2.5_ has significant impacts on human health and the economy (10–12), few studies have investigated the contributions of ACC and NV of human smoke exposure, not just fire activity, in the western United States (13). ACC influences wildfire emissions and smoke concentrations by altering wildfire activity, which includes the frequency, intensity, and severity of wildfires, as well as by altering atmospheric circulation and dynamics that affect wildfire smoke transport. For example, warming surface temperatures can dry out vegetation, increasing wildfire intensity or severity (4, 14). NV, driven by internal processes within the climate system, may also affect wildfire activity and ambient environment (15, 16). Improving our knowledge of the relative importance of these drivers of unhealthy smoke concentrations could spur greater action to address ACC and would focus efforts of fire management on specific regions where wildfires are most likely to affect human health downwind.

**Fig. 1.**
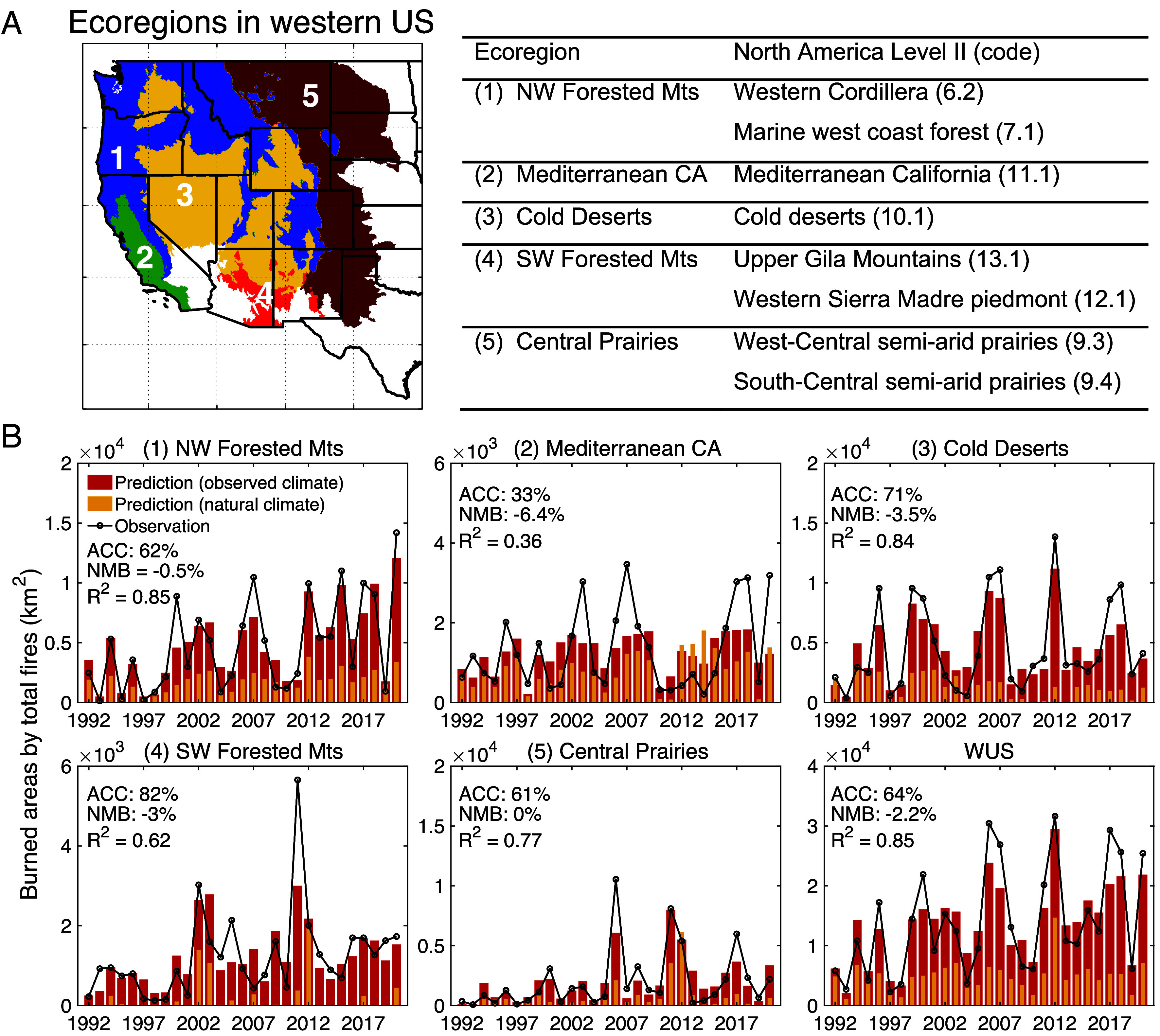
(*A*) Map of the five ecoregions in western United States used in this study. The table describes the different ecosystems, which are aggregated from data from the Environmental Protection Agency. (*B*) Timeseries of annual total burned area from 1992–2020 in five ecoregions in the western United States, as well as over the West as a whole. Black curves represent observed burned areas, including both lightning-ignited and human-ignited fires, from the FPA-FOD dataset. Colored bars show the predicted timeseries of annual burned area, calculated for observed (red) and “natural” (orange) conditions. The western United States (WUS) represents the sum of burned area in five ecoregions. Percent contributions of anthropogenic climate change (ACC) to the total predicted area burned are shown inset. The NMB and *R^2^* compare the predictions using observed climate conditions to the FPA-FOD dataset. Range of values on y-axes varies among panels.

Studies have found that climate change has been a major driver of fire weather and fuel aridity in the western United States, explaining a large portion (50 to 70%) of the interannual variability and increasing trend of wildfire burned area since the 1980s (2, 5, 17–19). Using fuel aridity as a proxy for burned area, Abatzoglou and Williams (4) suggested that ACC is responsible for doubling the trend in cumulative burned area in forested regions from 1984 to 2015. Beyond forested areas, other ecoregions have also experienced frequent wildfires in recent years, accounting for 60% of total burned area across the western United States from 1992 to 2020 (Fig. 1*A* and *SI Appendix*, S1). However, the extent to which ACC and NV contribute to wildfires in these other ecoregions remains largely unquantified. Complicating the issue is evidence that the meteorological influence on wildfire activity varies by ecoregion (20). In addition, the western United States is prone to both lightning- and human-ignited fires, each with fundamentally different characteristics. Here, human-ignited fires include those caused by power lines, campfires, smoking, debris burning, equipment use, and arson (21). Previous studies have shown that lightning-ignited fires tend to be more intense and larger in size than fires related to human activity, at least in some regions, and typically occur in summer and fall (June–October), while human-ignited fires can occur at any time of the year (22–24). These differences make it challenging to quantify the sensitivity of wildfires to different drivers.

Recent studies have explored how wildfires have affected present-day air quality and will likely degrade air quality in the future. For example, studies using air quality observations from monitoring stations and satellites have shown that wildfire smoke may now be the leading cause of poor air quality in the United States, with especially high concentrations in the western United States over the last decade (8, 9, 25). Recent work by Law et al. (13) demonstrates that ACC has significantly increased fire-related PM_2.5_ and related premature mortality across the United States from 2006 to 2020. In addition, studies have estimated future wildfire emissions using statistical models or process-based, dynamic vegetation models (20, 26–29). By applying projected fire emissions to offline chemical transport models, researchers have quantified the impact of climate change on wildfire smoke concentrations. In general, such studies have projected large increases in smoke concentrations across the western United States by 2050 or 2100, relative to the present-day (20, 26, 28–30). For example, over the Pacific Northwest, PM_2.5_ pollution could double or triple during the fire season due to increased wildfire activity by 2100, depending on the greenhouse gas emission pathway (28).

In this study, we investigate the extent to which ACC has already contributed to the increasing trends in wildfire smoke concentrations across the western United States from 1992 to 2020. We build on previous work by constructing detailed, ecoregion-dependent models of area burned, by considering lightning- and human-ignited fires separately, and by relying on a chemical transport model to calculate smoke concentrations. First, we divide the western United States into five ecoregions (31) and quantify the sensitivity of burned areas in these ecoregions to variations in climate variables. More specifically, we develop individual Gaussian Process Regression (GPR) models to establish relationships between observed burned areas from lightning- or human-ignited fires and a suite of possible drivers, including meteorological variables and the normalized difference vegetation index (NDVI) across the five ecoregions in the western United States. GPR is a machine learning algorithm based on a nonparametric Bayesian approach that is able to model highly nonlinear systems and provide robust predictive performance with limited data points (*SI Appendix*) (32). The resulting timeseries of predicted area burned in each ecoregion are referred to here as the “observed scenarios.” We consider the fire activity through the entire year to capture wildfire activity from both ignition sources. As noted above, human-ignited fires can occur year-round, significantly extending the typical fire season (22, 23). To capture the full spectrum of fire activity and ensure a consistent modeling approach across ignition sources, we develop the GPR models using annual means of climate and vegetation variables, consistent with the practice in previous studies of climate–fire relationships (20, 33, 34). This approach also allows us to account for antecedent climate and vegetation conditions that can influence fuel conditions and subsequent fire activity (18, 33–35). For example, one study found that winter and spring meteorological conditions could explain more than half of the interannual variability of summertime burned area in the western United States (18).

Second, we distinguish the signal of ACC from NV in the observed meteorology by using ensemble simulations from the sixth phase of the Coupled Model Intercomparison Project (CMIP6) (4, 5, 36, 37) (*Methods*). We generate a “natural” climate scenario by first estimating the long-term ACC signals from the CMIP6 and then subtracting these signals from the observed meteorology. We apply the natural climate scenario to the GPR models for lightning- and human-ignited fires in each ecoregion, yielding timeseries of expected burned area in a world unaffected by ACC over time. The predicted burned areas under both observed and natural climate scenarios are then used to quantify the ACC contribution to burned areas and to estimate natural fire emissions, following the framework of Global Fire Emission Database version 4.1 with small fires (GFED4.1s) (38). Finally, using the chemical transport model GEOS-Chem (39), we simulate long-term surface smoke concentrations across the western United States, driving the model with original fire emissions from GFED4.1s and estimated natural fire emissions under observed and natural climate scenarios. The difference between these two scenarios represents the smoke concentrations traced to ACC. In this way, we distinguish the effects of ACC from NV on smoke concentrations and quantify with greater confidence the magnitude that this deleterious consequence of a warming climate has already had across the western United States.

## Results

### Historical Trends of Lightning-Ignited and Human-Ignited Fires and Their Drivers Across the Western United States.

Our analysis of the Fire Program Analysis-Fire Occurrence Database (FPA-FOD) reveals that the contributions of lightning- and human-ignited fires to total burned area vary by ecoregion (*Methods* and *SI Appendix*). During the last three decades, lightning-ignited fires dominate fire activity in forested areas (NW and SW Forested Mts) and shrublands (Cold Deserts), accounting for 72% (NW Forested Mts), 75% (SW Forested Mts), and 62% (Cold Deserts) of total burned areas (*SI Appendix*, Figs. S1 and S2*C*). Human-ignited fires contribute 86% to total burned areas in southern California (Mediterranean CA) and 55% to semiarid prairies (Central Prairies) (*SI Appendix*, Figs. S1 and S2*D*); these regions are mostly covered by crops and grassland. Both lightning- and human-ignited fires show large interannual variability from 1992 to 2020 (*SI Appendix*, Fig. S1). We find that area burned by lightning-ignited fires has significantly increased in the NW Forested Mts from 1992 to 2020, with a Theil-Sen estimated slope of 0.48 σ decade^−1^ (σ represents the SD of annual burned areas in the given ecoregion), and in the SW Forested Mts at 0.54 σ decade^−1^ (*P* < 0.05). Significant increasing trends in human-ignited fires also occur in the NW Forested Mts, Cold Deserts, and Central Prairies, with Theil-Sen slopes of 0.47 σ decade^−1^ (NW Forested Mts), 0.31 σ decade^−1^ (Cold Deserts), and 0.23 σ decade^−1^ (Central Prairies) (*P* < 0.05). Other trends in burned area by lightning- or human-ignited fires are not significant in their respective ecoregions. Over the entire western United States, however, total burned areas of both lightning- and human-ignited fires increase significantly by 0.52 σ decade^−1^ (*P* < 0.05), with these increases dominated by fires occurring in the NW Forested Mts.

Comparing the GPR predictions with the FPA-FOD dataset, we find that meteorology and vegetation can explain 36 to 85% of the interannual variability in total burned areas across the ecoregions in the western United States (Fig. 1*B*). Here, the predicted total burned areas in each ecoregion are derived by summing the output from the GPR models for lightning- and human-ignited fires in that ecoregion. The key predictors include daily maximum air temperature ($T_{\max}$); moisture variables such as vapor pressure deficit (VPD), relative humidity (RH), specific humidity, and daily accumulated precipitation; and normalized difference vegetation index (NDVI). NDVI, derived from daily satellite observations, indicates the density and greenness of vegetation and can serve as an effective proxy for wildfire fuel loads.

For lightning-ignited fires (*SI Appendix*, Fig. S3), the GPR models accurately reproduce mean burned areas in four ecoregions, with normalized mean biases (NMBs) ranging from −4.2 to +2.6%, except for Mediterranean CA, where the mismatch is larger (NMB = −19.4%). The GPR models explain 79% (NW Forested Mts), 89% (Cold Deserts), and 64% (Central Prairies) of the variance in burned areas, revealing that in these regions meteorology and vegetation control a large portion of interannual variability in lightning-ignited fires. In the SW Forested Mts, however, lightning-ignited fires appear to be driven by other environmental or human factors, as the meteorology and vegetation variables chosen here account for only 44% of the interannual variability in burned areas. The GPR model performs poorly in capturing the variability of burned areas of lightning-ignited fires in the Mediterranean CA, where such fires are small and contribute only 14% to total burned areas.

For human-ignited fires (*SI Appendix*, Fig. S4), the GPR models slightly underestimate the mean burned areas in all regions with NMBs of −8 to −0.4%. As with lightning-ignited fires, meteorology and vegetation also greatly control the extent of burned areas of human-ignited fires, contributing 92% (NW Forested Mts), 64% (SW Forested Mts), and 85% (Central Prairies) to the interannual variability. In the Mediterranean CA and Cold Deserts, the GPR models explain ~40% of the variance in burned areas. This suggests that more complex anthropogenic factors, such as the extent and management of grazing and agricultural land, have a strong influence on the burned area extent of human-ignited fires.

Despite these discrepancies in interannual variability, the small NMBs show that our GPR models can nonetheless capture the total amount of burned areas from both lightning- and human-ignited fires over the last three decades. However, the GPR models do systematically underestimate burned areas by 19% across the western United States during extreme fire years, which we define as those years above the 80th percentile of observed annual burned areas from 1992 to 2020 for each ecoregion. These underestimates vary by ecoregion, with NMBs of −14% (NW Forested Mts), −48% (Mediterranean CA), −21%, (Cold Deserts), −25% (SW Forested Mts), and −24% (Central Prairies). Such underestimates at the high end of the distribution are not uncommon in machine learning regressions (40).

To better understand the role of individual predictors and their variability, we quantify the relative contribution of each predictor to burned area predictions by ignition source across five ecoregions (*Methods*). We find that the contributions of these predictors vary substantially by ecoregion and ignition source (*SI Appendix*, Fig. S5). For lightning-ignited fires, moisture variables (e.g., VPD, minimum or maximum RH, or specific humidity) are the most influential predictors across all ecoregions. Air temperature also plays a prominent role in the NW Forested Mts, Cold Deserts, and Central Prairies. For human-ignited fires, NDVI emerges as a key driver along with moisture variables, particularly in the Cold Deserts, NW Forested Mts, and Mediterranean CA. These differences likely reflect variations in climate regimes, fuel load, and ignition sources. In forested ecoregions, fire activity is more sensitive to climate as fuels are typically abundant. In contrast, in arid and semiarid ecoregions, fire activity is more limited by fuel availability. Thus, NDVI becomes a dominant driver, especially for human-ignited fires, which occur when sufficient vegetation is available.

We next assess the extent to which GPR model performance is driven by interannual variability versus long-term coincident trends. To that end, we conduct a sensitivity experiment by detrending both predictors and response variables in those ecoregions with significant trends in burned areas. We remove the linear trends from climate, vegetation, and burned area variables, and retrain the GPR models using these detrended datasets. Despite the removal of long-term trends, the GPR models retain strong predictive power for human-ignited fires in the NW Forested Mts (*R^2^* = 0.7), Cold Deserts (*R^2^* = 0.68), and Central Prairies (*R^2^* = 0.79), and for lightning-ignited fires in the NW Forested Mts (*R^2^* = 0.77) (*SI Appendix*, Fig. S6). The *R^2^* value decreases from 0.44 to 0.33 in the SW Forested Mts (*SI Appendix*, Fig. S6*D*), where the relationship between predictors and lightning-ignited fires is more complex. These results demonstrate that a substantial portion of the explanatory power derives from capturing interannual variability rather than from correlations in persistent long-term trends, supporting the robustness of the GPR modeling framework.

### Contributions of ACC to Burned Areas Across the Western United States.

Anthropogenic forcing leads to a warmer and drier climate across the western United States with notable regional variations in air temperature, VPD, and precipitation (*SI Appendix*, Figs. S7 *A*-*C* and S8). We find that anthropogenic forcing results in increasing trends in the ensemble mean for $T_{\max}$ of 0.48 ± 0.0063 K decade^−1^ (95% CI) and for VPD of 0.025 ± 0.0011 kPa decade^−1^ (95% CI) across the entire western United States from 1992 to 2020 (*SI Appendix*, Fig. S7 *A* and *B*). These increasing trends for both $T_{\max}$ and VPD, which we define as ACC signals, are significant across multiple CMIP6 models, with ranges of 0.06 to 0.76 K decade^−1^ for $T_{\max}$ and 0.004 to 0.05 kPa decade^−1^ for VPD. The trend in the ensemble mean for daily accumulated precipitation over the entire western United States over this period is +0.025 ± 0.0033 mm decade^−1^ and is not statistically significant (*SI Appendix*, Fig. S7*C*). *SI Appendix*, Fig. S8 *A*–*C* shows the spatial distributions of ACC signals for these variables averaged over 1992 to 2020 and the significance of the ACC trends in each grid cell. Results indicate that the increases in $T_{\max}$ and VPD vary by ecoregion in the western United States. The central and southwestern regions experience the greatest increases in $T_{\max}$ and VPD, reaching 1.7 K and 0.18 kPa, respectively (*SI Appendix*, Fig. S8 *A* and *B*). However, anthropogenic forcing has contrasting effects on simulated precipitation in different regions, with these effects reaching statistical significance in most grid cells (*SI Appendix*, Fig. S8*C*). Such forcing reduces average daily accumulated precipitation by 0.05 to 0.08 mm in the northern California and coastal area of Oregon but slightly increases it by 0.02 to 0.05 mm in central areas.

Under the natural climate scenario that excludes ACC signals, burned areas predicted by the GPR models account for only 35% of those in the FPA-FOD dataset in the whole western United States from 1992 to 2020. Fig. 1*B* compares the total burned areas predicted under the natural climate scenario with those predicted using the observed climate scenario in five ecoregions over this period. The differences in predicted burned areas under the two climate scenarios represent the contribution of ACC to the trends in total burned areas. In most years, ACC amplifies the predicted area burned compared to that in the natural scenario. Our results suggest that ACC contributes 33 to 82% to total burned areas in five ecoregions during the last three decades; this range reflects the different responses of lightning- and human-ignited fires to ACC.

We further find that lightning-ignited fires are more sensitive to ACC across most ecoregions, with ACC contributing 64 to 92% to the burned areas of these fires (*SI Appendix*, Fig. S3). The impacts of ACC on lightning-ignited fires are larger in forest and shrubland ecoregions, with ACC contributions of 69% (NW Forested Mts), 79% (Cold Deserts), and 92% (SW Forested Mts). Human-ignited fires are somewhat less affected by ACC, with 35 to 64% of human-ignited burned areas due to ACC in five ecoregions (*SI Appendix*, Fig. S4). ACC yields the lowest contributions (35%) to burned area of human-ignited fires in the Mediterranean CA, the region that is most densely populated and characterized by a significant proportion of agricultural land. Finally, we find that the dominant ACC-driven climatic factors of burned area vary by ecoregion (*SI Appendix*, Fig. S9). In densely forested areas (NW and SW Forested Mts), both lightning- and human-ignited fires are most sensitive to increasing VPD; in grassland and shrub areas (Mediterranean CA, Cold Deserts, and Central Prairies), both kinds of fires are most sensitive to increasing $T_{\max}$. Furthermore, we also find different sensitivities between lightning- and human-ignited fires to the identical ACC perturbations in VPD and $T_{\max}$ (23).

### Contributions of ACC to Fire Emissions and Smoke Concentrations Across the Western United States.

**Fig. 2.**
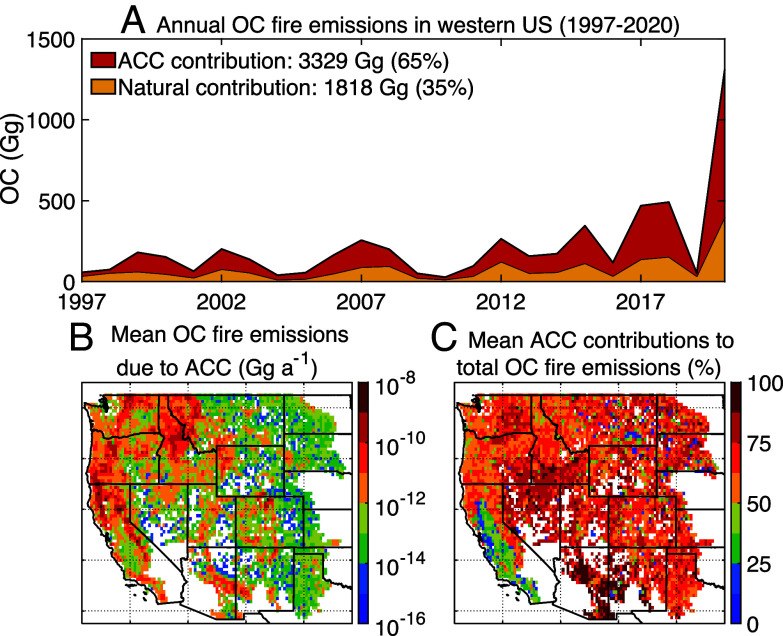
(*A*) Timeseries of annual total fire emissions of organic carbon (OC), the primary component of smoke PM_2.5_, under the observed climate conditions (sum of red and yellow areas), as reported in the GFED4.1s inventory for the western United States from 1997 to 2020. Also shown are the OC fire emissions under the natural climate scenario (yellow areas) and those emissions attributed to ACC (red areas). Values inset represent the OC fire emissions in Gg and the percent contributions to total OC fire emissions due to natural variability (NV) and to ACC, averaged over the time period. The *Bottom* row shows the spatial distributions of (*B*) mean annual OC fire emissions due to ACC, averaged from 1997 to 2020, and (*C*) mean ACC percent contributions to total OC fire emissions in each grid cell.

We compare the time series of annual fire emissions of organic carbon (OC) under the observed and natural climate scenarios in the western United States from 1997 to 2020 (*Methods*). In the GFED4.1s inventory, annual fire emissions of OC significantly increase by 11 Gg a^−1^ (*P* < 0.05) over 1997 to 2020 in the western United States, with cumulative emissions of 5.1 × 10^3^ Gg. Under natural climate conditions, the small increase in annual OC fire emissions is not statistically significant, with total emissions of 1.8 × 10^3^ Gg summed over all years, or approximately one-third of the total in the observed scenario (Fig. 2*A*). The interannual variability in OC fire emissions is driven by natural climate variability. The difference in annual OC fire emissions between GFED4.1s and the natural scenario represents the ACC contribution to these emissions, ~65% of total fire emissions. We find that for the entire western United States, ACC explains ~68% of the increasing trend in OC emissions from 1997 to 2020. Across the five ecoregions, ACC contributes 26 to 76% to OC fire emissions (*SI Appendix*, Fig. S10), proportional to the ACC contributions to total burned areas.

Our results indicate that large fire emissions driven by ACC occur in the forested regions along the Coast Ranges, the Sierra Nevada, the Cascades, and in northern Idaho, accounting for ~60% of current fire emissions in the western United States from 1997 to 2020 (Fig. 2*B*). The mean ACC contributions range from 50 to 75% over the last two decades. The region where fire emissions are most affected by ACC (~90%) is in the Upper Gila Mountains, which stretch across Arizona and New Mexico (Fig. 2*C*). In southern California, where emissions are dominated by human-ignited fires, the ACC contributions to these emissions are less than 50% (Fig. 2*C*). The rest of western United States is covered mainly by savanna and shrubland, and we find that fire emissions account for only 2% of total emissions over the time period, due to low fuel loads and low emission factors for these land types.

To quantify the contributions of ACC and NV to long-term smoke concentrations, we use the fire emissions under the two climate scenarios (observed and natural) as input to GEOS-Chem (*Methods*). *SI Appendix*, Table S1 shows the model configurations for three sensitivity experiments. The control experiment (CTL) and natural experiment (NAT) simulate PM_2.5_ concentrations from both fire and nonfire sources under observed and natural climate scenarios. The magnitudes of nonfire sources (e.g., industry or dust mobilization) are the same in both scenarios. The background experiment (BKG) simulates only PM_2.5_ concentrations from nonfire sources. Even though most smoke PM_2.5_ is generated from August through October in the western United States, we focus on annual means to better compare with nonfire PM_2.5_, which may have a different seasonality (*SI Appendix*, Fig. S11).

Based on the BKG simulation, we find that the background concentrations of PM_2.5_ from nonfire sources decreases by 44% averaged over the western United States from 1997 to 2020, mainly due to the reduction in emissions of air pollutants under the Clean Air Act (41) (*SI Appendix*, Fig. S11*A*). In contrast, annual mean concentrations of total PM_2.5_ plateau and then reverse over the most recent decade. Here, the difference in annual mean concentrations between the CTL and the BKG experiments represents the smoke PM_2.5_ contributed by wildfires under observed climate conditions (*SI Appendix*, Fig. S11*D*). Our results show that the annual mean concentrations of smoke PM_2.5_ averaged across the western United States range from 0.2 to 4.0 µg m^−3^ from 1997 to 2020, with an average 0.73 µg m^−3^. Results further highlight a significant increasing trend in smoke PM_2.5_ of 0.2 µg m^−3^ a^−1^ (*P* < 0.05) from 2010 to 2020. The contribution of smoke PM_2.5_ to total PM_2.5_ exhibits large interannual variability from 1997 to 2020, ranging from 5 to 62%, again with an increasing trend over the most recent decade (*SI Appendix*, Fig. S12). During the extreme wildfire year of 2020, smoke PM_2.5_ contributes 62% to total PM_2.5_. We also find similar trends in smoke organic aerosol (OA) and smoke black carbon (BC) during this period (*SI Appendix*, Fig. S11).

We find that ACC contributes nearly half (49%) to mean concentrations of smoke PM_2.5_ over the western United States from 1997 to 2020 (*SI Appendix*, Fig. S11*D*), with annual mean concentrations ranging from 0.04 to 2.7 μg m^−^^3^ averaged across the western United States and a multiyear mean of 0.4 µg m^−3^. These values are determined by differencing the concentrations under the observed and natural scenarios. Under the natural climate scenario, annual mean concentrations of smoke PM_2.5_ range from 0.1 to 1.3 µg m^−3^ averaged across the western United States from 1997 to 2020. During the most recent decade (2010 to 2020), ACC accounts for 58% of the increasing trend in smoke PM_2.5_, and the contribution of ACC to mean smoke PM_2.5_ concentrations rises to 54%.

**Fig. 3.**
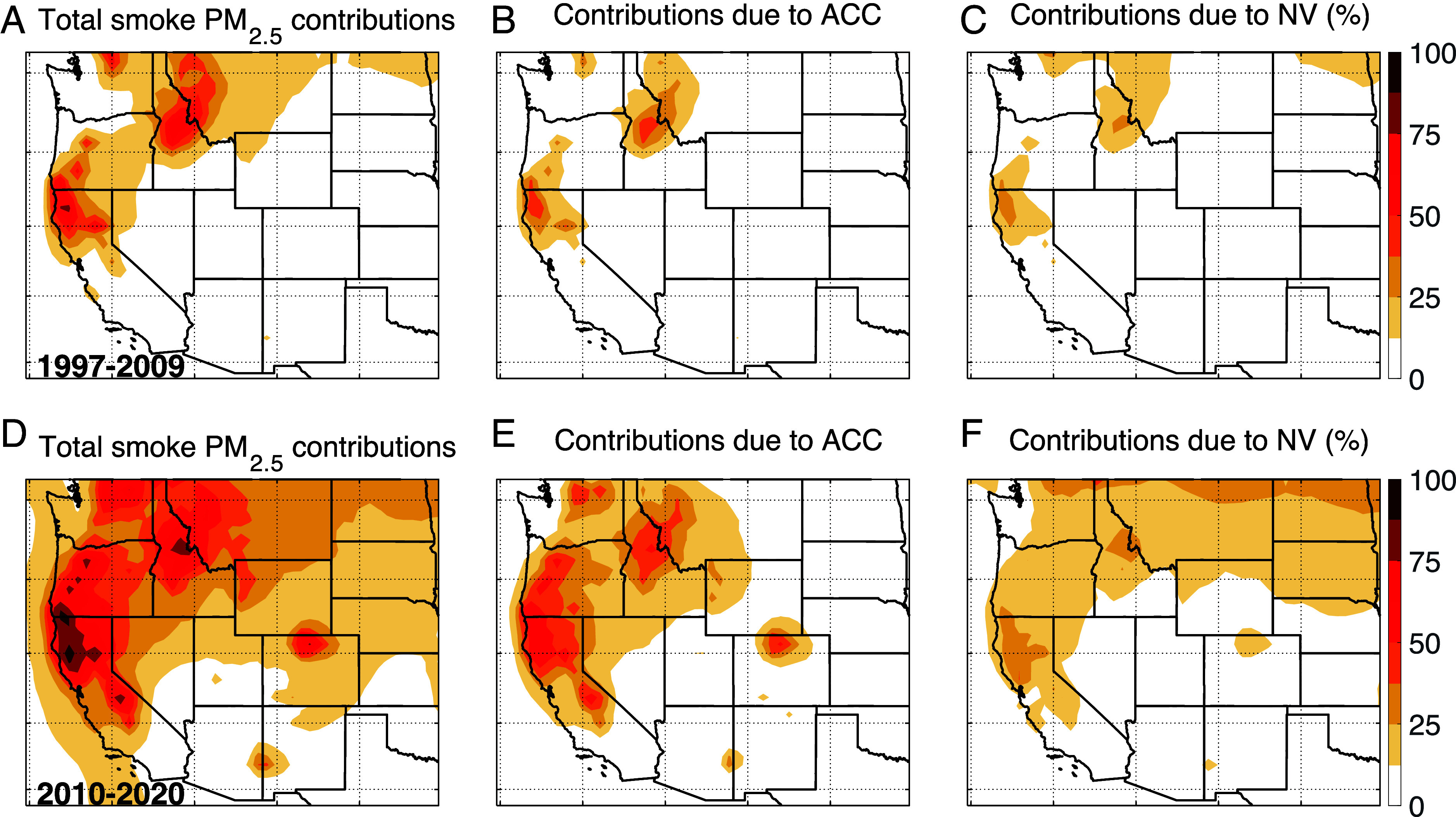
Calculated percent contributions of smoke PM_2.5_ to total surface PM_2.5_ for 1997 to 2009 and for 2010 to 2020. The *Top* panels show the spatial distributions of the contributions of all smoke PM_2.5_ to total PM_2.5_ (*A*) and the contributions of smoke PM_2.5_ to total PM_2.5_ attributed to ACC (*B*) and NV (*C*) for 1997 to 2009. The *Bottom* panels show the contributions of all smoke PM_2.5_ to total PM_2.5_ (*D*) and the contributions of smoke PM_2.5_ to total PM_2.5_ attributed to ACC (*E*) and NV (*F*) for 2010 to 2020.

We next examine more closely the spatial distributions of the percent contributions of smoke PM_2.5_ to total PM_2.5_ in the western United States over two periods: from 1997 to 2009 and from 2010 to 2020 (Fig. 3). Our results show that wildfire smoke is especially abundant in northern California, Oregon, northern Idaho, and western Montana, where smoke PM_2.5_ accounts for 25 to 80% of total PM_2.5_ from 1997 to 2009 (Fig. 3*A*). Over the rest of the western United States, the contributions of smoke PM_2.5_ to total PM_2.5_ are less than 10%. We find that both ACC and NV drive the smoke PM_2.5_ during this period, with 10 to 50% traceable to ACC (Fig. 3*B*). However, during the more recent period of 2010 to 2020, the impacts of smoke PM_2.5_ expand to most of the states of northwestern United States (Fig. 3*D*), and the contributions of smoke PM_2.5_ to total PM_2.5_ increase to 50 to 95% over northern California, Oregon, Washington, and Idaho. Our results indicate that this 2010–2020 enhancement of smoke PM_2.5_ is mostly driven by ACC, accounting for 25 to 66% of total PM_2.5_ concentrations across these regions (Fig. 3*E*). The rest of the smoke PM_2.5_ concentration is attributed to NV, with percent contributions of 10 to 30% (Fig. 3*F*).

**Fig. 4.**
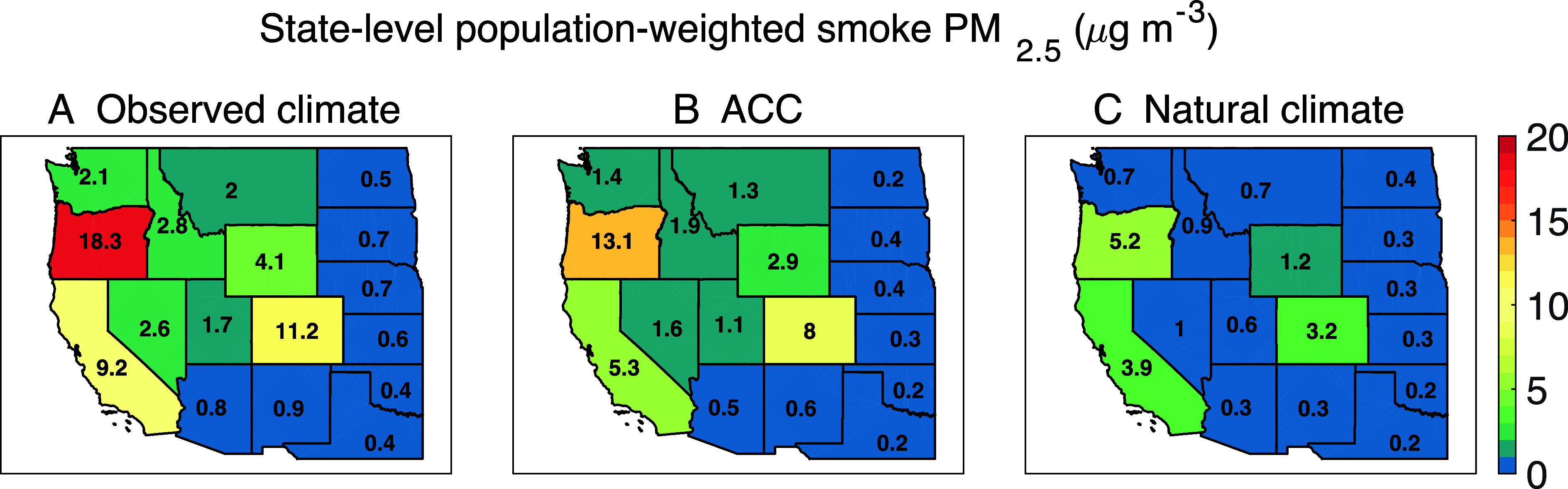
Simulated annual mean population-weighted smoke PM_2.5_ concentrations across the western United States in 2020 under different climate scenarios. Units are µg m^–3^. The *Left* (*A*) panel shows the state-level population-weighted smoke PM_2.5_ concentrations under the observed scenario. The *Center* (*B*) and *Right* (*C*) panels show the state-level population-weighted smoke PM_2.5_ concentrations attributed to ACC and NV. The values *Inset* represent the simulated population-weighted smoke PM_2.5_ concentrations for each state. Large values reveal those states where large numbers of people are exposed to high levels of smoke PM_2.5_. In the extreme fire year 2020, the ACC contributions to population-weighted smoke PM_2.5_ concentrations average over 60% in most western states.

Since much of the western United States is sparsely populated, it is instructive to examine population-weighted smoke concentrations (8) (*SI Appendix*). This metric allows policymakers and stakeholders to more clearly pinpoint where smoke has become a public health concern, especially smoke driven by ACC. To that end, we calculate the state-level population-weighted smoke PM_2.5_ concentrations and attribute them to NV and ACC for the extreme fire year 2020 (Fig. 4). We find that Oregon, Colorado, and California emerge as the states with the greatest population-weighted smoke PM_2.5_ concentrations attributable to ACC in that year, with concentrations of 13.1 µg m^−3^, 8.0 µg m^−3^, and 5.3 µg m^−3^, respectively. To pinpoint areas where wildfire smoke affects the largest populations, we also multiply the gridded smoke PM_2.5_ concentration by the ratio of the population in each grid cell to the total population in the western United States for these representative years: 2000, 2005, 2010, 2015, and 2020 (*SI Appendix*, Fig. S13). Examining individual years illustrates how ACC may have shaped different fire seasons. We acknowledge, however, that accurate attribution of ACC to individual years requires more sophisticated modeling beyond the scope of this study. Here, we find that from 2000 to 2010, annual population-weighted smoke PM_2.5_ concentrations are 0.15 to 0.2 µg m^−3^ over the entire western United States, with only 20 to 40% of these concentrations attributed to ACC. However, the population-weighted concentrations increase to 0.61 and 5.2 µg m^−3^ in 2015 and 2020, respectively, with ACC contributions rising to 44 and 62%. Our results indicate that populations in urban and suburban areas in California, western Oregon, Colorado, and Washington are most vulnerable to the threat of wildfire smoke. In the extreme fire year 2020, we find that populated-weighted smoke PM_2.5_ concentrations average 9.2 µg m^−3^ over California, 11.2 µg m^−3^ over Colorado, and 18.3 µg m^−3^ over Oregon. Also in that year, a hotspot of increased population-weighted smoke concentrations appears along the Front Range urban corridor in Colorado. Compared to NV, ACC emerges as the dominant driver in 2020, contributing 60 to 70% of the population-weighted smoke concentration.

## Conclusions and Discussion

By quantifying the ACC impact on wildfires and smoke concentrations across the western United States, our work builds on previous work documenting the influence of ACC on area burned in western US forests in recent decades (4, 7). We find that the influence of ACC on area burned largely depends on type of ecoregion and fire ignition source. The ACC contributions to burned areas in forested regions (NW and SW Forested Mts) range from 62 to 82% during 1992 to 2020, greater than the approximately 50% contribution reported by Abatzoglou and Williams (4) for similar forested areas, summed over 1984–2015. The increase in ACC contributions may reflect the influence of recent large fires in the western United States. In relatively sparsely populated regions dominated by grassland or shrubs (Cold Deserts and Central Prairies), ACC accounts for 61 to 71% of burned areas. These ecoregions, including many national forests, parks, wilderness areas, and areas with highly flammable invasive species, are primarily affected by lightning-ignited fires that have been allowed to burn with less human intervention since the 1970s (42). Thus, ACC has a significant impact in these ecoregions. Even in the densely populated Mediterranean California region (Mediterranean CA), where many anthropogenic factors influence fire activity (30), we find that ACC accounts for 33% of area burned over 1992–2020. The lower ACC contribution in the Mediterranean CA is likely due to the high percentage of human-ignited fires (over 70%), which are often less intense, smaller in size, or more accessible, and can occur under higher fuel moisture conditions, compared to lightning-ignited fires (23). Human-ignited fires are thus more likely to be rapidly suppressed or managed. Previous studies have also demonstrated that human ignitions triple the length of the wildfire season (22, 23). These findings imply that the occurrence of human-ignited fires may be less dependent on weather and fuel conditions than lightning-ignited fires and so less sensitive to ACC.

Given the seasonal dynamics of wildfires, climate, and vegetation, ACC contributions to burned areas may vary during the fire season. To evaluate the seasonality impacts on ACC attribution, we develop additional GPR models using fire-season (May–October) data for both burned area and the predictors. We then estimate the ACC contributions to burned areas following the same attribution framework described in the *Methods*. Compared to original annual-scale results, we find that ACC contributions to fire-season burned areas vary across ecoregions ranging from 20 to 79%, but the overall ACC contribution across the entire western United States remains stable, estimated at 65% (*SI Appendix*, Fig. S14). However, substantial regional differences in ACC contributions emerge due to varying sensitivities of fire activity to fire-season climate conditions and ACC signals. To explore these sensitivities, we use Partial Dependence Plots (*SI Appendix*, Fig. S15) to examine relationships between the predictors and burned area across ecoregions. Our results show that lightning-ignited fires generally respond linearly to key climate variables in both annual and fire-season models, whereas human-ignited fires exhibit stronger nonlinearities. The predictor-burned area relationships differ between annual and fire-season models, contributing to the observed regional differences in ACC attribution (*SI Appendix*).

By estimating the ACC contribution to observed PM_2.5_ trends in the western United States from 1997 to 2020, our work extends previous studies that have identified the increasing impact of smoke PM_2.5_ on air quality, such as Burke et al. (9). A recent study estimated that 25 to 60% of the average annual wildfire PM_2.5_ in California, Oregon, Washington, Idaho, and Montana can be attributed to climate change from 2006 to 2020 (13). Consistent with such studies, our results reveal an increase in smoke PM_2.5_ around 2010, reversing a decreasing trend in total PM_2.5_ across the western United States. We find that 25 to 66% of total PM_2.5_ concentrations across the western United States during 2010–2020 can be attributed to the ACC influence on fire emissions, with 58% of the increasing trend in smoke PM_2.5_ linked to ACC. Relatively remote areas in Idaho and northern California experience the greatest ACC impacts on smoke PM_2.5_, with as much as 40 to 66% of total PM_2.5_ attributable to ACC during this period (Fig. 3). However, densely populated areas such as the Central Valley in California and the Willamette Valley in Oregon exhibit high levels of population-weighted smoke concentrations (*SI Appendix*, Fig. S13), making clear the large threat that ACC poses for human health.

We base our work on several assumptions. First, we assume that the interannual variability in burned areas in the western United States is solely driven by meteorology and vegetation. However, other factors, such as changing land use and land management practices, as well as the history of fire suppression policies and prescribed burning, also contribute to the interannual variability, especially in areas outside national forests and parks (43, 44).

Second, our attribution framework relies on the estimation of ACC signals in climate variables, and we acknowledge the uncertainties associated with our ACC signal removal approach. We use only a single realization from each CMIP6 model rather than realization means, which yields somewhat noisier timeseries of climate variables for ACC signal estimation (*Methods*). We also assume that the climate sensitivity averaged across CMIP6 models approximates the true climate sensitivity in the real world. However, climate sensitivity to increasing greenhouse gases varies considerably among models (45), which may introduce biases in the derived ACC signals and NV in climate variables (*SI Appendix*, Fig. S7 and *Methods*). Finally, alternative robust approaches for estimating and removing the ACC signal, such as regression-based methods, may lead to different ACC attribution results for burned area and wildfire smoke. Assessing how attribution outcomes depend on the choice of ACC signal estimation method represents an important direction for future work.

Third, we assume that fire emissions outside the western United States are driven by NV, which means that our interpretation of smoke concentrations in the far northern states—eastern Montana, North Dakota, and South Dakota—comes with a caveat (Fig. 3*F*). While our model framework attributes 10 to 30% of smoke PM_2.5_ concentrations in this region to NV, we speculate that much of this smoke can likely be traced to long-range transport of wildfire smoke from Canada.

Finally, we assume that ACC influences the wildfire activity and smoke concentrations solely through its impacts on meteorological variables. Another potential pathway through which ACC may affect wildfires and smoke concentrations involves its influence on vegetation. Since fully dynamic land cover changes are not considered in the CMIP6 simulations used here, we apply the same time series of NDVI to both observed and natural climate scenarios in the regression models. In this way, we assume that the interannual variability in NDVI is entirely attributable to the NV of climate. In addition to NDVI, we also evaluate vegetation optical depth (VOD), a proxy for vegetation moisture, as an input variable in the GPR models (*SI Appendix*). Results show that including VOD significantly improves the prediction of interannual variability in burned area in one ecoregion, Mediterranean CA (*SI Appendix*, Fig. S16). However, since we are unable to explicitly quantify the ACC impacts on either NDVI or VOD, our estimates of ACC contributions to burned areas derived from this additional experiment remain broadly consistent with our original findings. Additionally, ACC may also affect smoke concentrations by altering atmospheric circulation and fire severity, which in turn influence the transport and dispersion of wildfire smoke. These complexities could be addressed in future research.

Exposure to ambient PM_2.5_ ranks first among the leading causes of disease worldwide (46) and smoke PM_2.5_ from wildfires thus represents a significant human health hazard (10, 11, 13). One study estimates that ACC led to approximately 15,000 wildfire PM-related deaths from 2006 to 2020 (13). While several studies have projected the influence of climate change on future smoke PM_2.5_ in the western United States (20, 26, 28), here we quantify the substantial effect that ACC has already had on smoke concentrations in recent decades (1997–2020). Understanding the specific contributions of ACC versus NV in driving smoke concentrations can help policymakers gain insights into where interventions may be most effective. For example, our study strengthens the case for aggressive and sustained land management in those regions where increasing wildfire activity generates the greatest smoke concentrations downwind (47). By highlighting increased smoke concentrations as a key consequence of a warming climate, this work provides additional impetus to develop more aggressive policies aimed at reducing greenhouse gas emissions.

## Methods

### Wildfire Burned Area Data.

We obtain the burned area data for lightning-ignited fires and human-ignited fires in the western United States during 1992 to 2020 from the Fire Program Analysis-Fire Occurrence Database (FPA-FOD) (48). This database provides the discovery date, a point location, burned area, and fire cause of each wildfire record reported by a consortium of federal, state, and local organizations. We examine a total of 734,879 records of wildfires, each classified as having “Natural,” “Human,” and “Missing/not specified/undetermined” causes. Prescribed fires are not included in the FPA-FOD. We analyze the wildfire records labeled “Natural” and “Human,” accounting for 33.5 and 58.3% of the total records. Records classified as having “Missing/not specified/undetermined” causes, about 8.2% of the total, are excluded in this study.

To validate the annual total burned area data from FPA-FOD, we use the satellite-derived burned area data from the Monitoring Trends in Burn Severity (MTBS) (49) (*SI Appendix*). MTBS relies on Landsat and Sentinel-2 prefire and postfire satellite imagery to drive the differenced normalized burn ratio and map burned area and severity at a spatial resolution of 30 m (49). MTBS includes the burned area boundary, burn severity, ignition date, location, and fire types of each record since 1984. The three fire types are “Wildfire,” “Prescribed Fire,” and “Unknown.” To compare with FPA-FOD, we use only the records classified as “Wildfire.” MTBS contains records of only those wildfires with burned area greater than 1,000 acres in the western United States, thus missing small fires (*SI Appendix*).

To assess the interannual trends in total lightning- and human-ignited fires, we use the Theil-Sen estimator (50, 51) to calculate the slopes and test the trend significance using the nonparametric Mann–Kendall algorithm (52, 53) with a significance level of 0.05. To compare the spatial distribution of the two datasets, we match the burned areas to align with a 0.25° × 0.25° spatial resolution.

### Climate Data and ACC Signals from CMIP6 Simulations.

Observed daily meteorological data from 1992 to 2020 are obtained from the gridMET dataset (54) and include maximum air temperature ($T_{\max}$), vapor pressure deficit (VPD), minimum and maximum relative humidity (${\mathrm{RH}}_{\min}$ and ${\mathrm{RH}}_{\max}$), specific humidity (SPH), accumulated precipitation (PRECIP), and wind direction (WD). The gridMET dataset has a spatial resolution of 1/24th degree (~4 km).

We rely on an ensemble of simulated meteorological output from CMIP6 to separate the long-term signals of ACC from NV. While the CMIP6 “historicalNAT” simulations are valuable for isolating natural climate variability, we did not use them in this study because they end in December 2014, limiting their use for assessing recent and extreme fire years. We follow the method described in Chen et al. (37), which defines the ACC signal as the multimodel ensemble mean of absolute differences between the 20-y moving averages of a particular variable (i.e., 1941–60, 1942–61, …, 2081–2100) and the mean value during the reference period (1921–1940). We construct an annual time series of each meteorological variable from the historical experiment (1920–2014) and the Shared Socioeconomic Pathway 5—Representative Concentration Pathway 8.5 (SSP5-8.5) experiment of the available CMIP6 models for 2015–2020. SSP5-8.5 does not differ significantly from the other pathways in this short, near-term period (55). For consistency, we use the first ensemble member simulation of each CMIP6 model driven by the same model configuration (r1i1p1f1) (56), consistent with the practice in previous attribution studies involving wildfire (13, 57). *SI Appendix*, Table S2 shows the availability of CMIP6 models for the meteorological variables selected for this study. We acknowledge that using a single realization of each model results in a noisier time-series of climate variables (*SI Appendix*, Fig. S17*A*). To evaluate the impact of this choice, we performed a sensitivity test in which we recalculate the $T_{\max}$ ACC signal by first averaging over all available realizations within each model, and then averaging across the realization-means of multiple models. The number of realizations available for $T_{\max}$ for each model is summarized in *SI Appendix*, Table S2.

Use of an ensemble of climate model outputs allows us to sample models with a range of strengths and biases (4, 5, 36, 37). *SI Appendix*, Fig. S17 illustrates the calculation of the ACC signal in $T_{\max}$ for the NW forested Mts. Although CMIP6 models differ substantially in their absolute values in $T_{\max}$ (*SI Appendix*, Fig. S17*A*), the derived ACC signals show rough agreement in both trend direction and magnitude (*SI Appendix*, Fig. S17*B*), providing some confidence in this approach. By relying on the departures from a reference period mean, rather than the absolute values of CMIP6 variables, we reduce the influence of model-specific biases. To mitigate the impact of high-frequency natural climate variability and better isolate the long-term anthropogenic signal, we apply a 20-y moving average to CMIP6 outputs. For the natural climate simulation, we subtract the ACC signal from the gridMET data to obtain the timeseries of NV in each meteorological variable (*SI Appendix*, Fig. S17*A*). These residuals represent the combined influence of internal variability and natural external forcings, including variations in solar irradiance and volcanic aerosols. The resulting timeseries of natural climate variables are then applied to GPR models to predict the burned areas unaffected by anthropogenic forcing.

*SI Appendix*, Fig. S17 also compares the ACC signals and NV in $T_{\max}$ from the single realization and realization-mean approaches. We find that the realization-mean approach reduces the noise in annual time series associated with internal variability in individual CMIP6 models (*SI Appendix*, Fig. S17*A*). However, the resulting ACC signals and natural variabilities in $T_{\max}$ from the two approaches are consistent (*SI Appendix*, Fig. S17), with mean difference ranging from 0.008 to 0.09 K in five ecoregions. This agreement is likely due to the application of the 20-y low-pass filtering, which reduces intrinsic variability and naturally forced variability from volcanic eruptions, solar irradiance changes, and other phenomena. When we apply the NV in $T_{\max}$ from the realization-mean approach to the GPR models, the resulting changes in ACC contributions are less than 3% across five ecoregions, which gives us confidence in our approach using single realizations.

To further evaluate the CMIP6 model outputs in reproducing historical climate variability, we compare the timeseries of key climate variables from CMIP6 simulations with gridMET observations over the western United States during 1992 to 2020 (*SI Appendix*, Fig. S7 *D*–*F*). The CMIP6 ensemble mean generally captures observed trends and magnitudes in $T_{\max}$ and VPD, supporting their use in ACC signal estimation. However, individual CMIP6 models diverge in simulating interannual variability, particularly for VPD and precipitation, resulting in systematic biases. Although our approach cannot entirely eliminate these biases, we adopted it for its simplicity in removing the low-pass filtered signal across all climate variables from the CMIP6 models, while retaining the naturally forced variability in the observations (4, 13, 37). Other approaches, such as regression-based removal, are also widely used and can yield valuable insights, though they rely on different assumptions and may result in ACC signals differing from those of our method (36, 58, 59).

We use the original monthly mean data from CMIP6 models, which have varying spatial resolutions. All model outputs are regridded to a common resolution of 1° × 1°. Since VPD is not directly provided by CMIP6, we calculate it using near-surface air temperature and near-surface relative humidity, following the method described by Zhuang et al. (5) (*SI Appendix*). Zhuang et al. (5) has demonstrated that VPD trends derived from monthly and daily data are highly consistent across the western United States, supporting our use of monthly data for long-term analysis.

### Gaussian Process Regression Model.

We develop the regression relationships between potential drivers and observed burned areas of lightning- and human-ignited fires in five ecoregions during 1992 to 2020 using the GPR, a machine learning algorithm based on a nonparametric Bayesian approach (32). The ecoregions are aggregated from the Level II Ecoregion data from the Environmental Protection Agency (Fig. 1*A*) (31). We choose the GPR model as it can model highly nonlinear systems effectively and achieve good predictive performance with fewer data points than other methods (32). In this study, all predictors for the GPR models consist of the annual mean values of observed climate data from the gridMET dataset and the NDVI derived from the NOAA polar orbiting satellites (Advanced Very High Resolution Radiometer and Visible Infrared Imaging Radiometer Suite) (60, 61), while the target variables are the annual total burned areas of lightning- or human-ignited fires obtained from FPA-FOD in each ecoregion. We build GPR models using domain-averaged variables at the ecoregion scale rather than near fire locations, representing the overall climate and vegetation conditions and mitigating the influence of short-term, localized changes in NDVI caused by extreme fire events. Use of observed data as predictors, rather than the CMIP6 output, avoids possible biases in the models and ensures that the interannual variability of the predictors matches that of the target variables. To select the predictors in each ecoregion, we build GPR models with different combinations of all variables and then select the one yielding the highest *R^2^* between the predicted and observed burned areas. *SI Appendix*, Table S3 shows selected predictors for lightning- and human-ignited burned areas in five ecoregions. We use 5-fold cross validation to evaluate the performance of the resulting GPR models. The resulting *R^2^* and root mean square errors (RMSEs) between the predictions from GPR models and FPA-FOD observations are also shown in *SI Appendix*, Table S3.

We use the SHapley Additive exPlanations (SHAP) values to interpret the relative importance of each predictor in the GPR models. SHAP assigns each predictor a contribution score based on its marginal impact on the model output and is widely used for interpreting machine learning models (62). For each year, we calculate SHAP values for each climate and vegetation variable and then average them over 1992 to 2020. We convert the mean absolute SHAP values into percentage contributions by normalizing them to sum to 100% within each model. This approach highlights the relative contribution of each predictor to burned area predictions and indicates how their contributions vary by ecoregion and ignition source (*SI Appendix*, Fig. S5).

To better understand the sensitivity of both lightning- and human-ignited fires to ACC contributions of each meteorological driver in five ecoregions, we conduct a set of sensitivity experiments in which we exclude the ACC signals one by one from each of the observed meteorological variables applied to the GPR models. We then recalculate the burned areas using the resulting input datasets. *SI Appendix*, Fig. S9 shows the individual and combined ACC contributions of meteorological variables to predicted burned areas of lightning- and human-ignited fires in each ecoregion.

### Fire Emissions for the Observed and Natural Climate Scenarios.

Total fire emission of a specific species in GFED4.1s is a function of dry matter burned, emission factors for different fire sources (grassland and savanna, woodland, deforestation and degradation, forest, agricultural waste burning, and peat fires), and the fractional contributions of each fire type in a grid cell. Dry matter burned is calculated by multiplying burned area by fuel consumption. Burned area data compiled for the GFED4.1s inventory are derived from the Moderate Resolution Imaging Spectroradiometer (MODIS) observations onboard the Terra and Aqua satellites (63); these data represent current burning levels under observed climate conditions. In GFED4.1s, fuel consumption statistics are estimated by the Carnegie-Ames-Stanford Approach (CASA) biogeochemical model (38). Emission factors for individual species in units of g species per kg dry matter burned are taken from published studies and vary with land cover (64–66). The GFED4.1s inventory, which has a spatial resolution of 0.25° × 0.25°, is available only from 1997 to 2020, and so all our smoke simulations cover this timeframe.

In this study, we rely on original data from the GFED4.1s as the fire emissions under the observed scenario, as the annual burned area data from the GFED4.1s inventory are in good agreement with the FPA-FOD data in each ecoregion (*SI Appendix*, Fig. S18), which in turn are in good agreement with results from the GPR models. To calculate the expected fire emissions under the natural climate scenario, we use our predicted burned area data under this scenario following the GFED4.1s framework described above. We estimate dry matter burned under the natural climate scenario based on the relationship between dry matter burned and burned area in the GFED4.1s. Our approach assumes the same spatial distribution of area burned as in GFED4.1s, but scales dry matter burned. *SI Appendix*, Fig. S19 illustrates the strong linear correlations (*R^2^* = 0.6 to 0.9) between the annual total dry matter burned and annual total burned areas from the GFED4.1s inventory in five ecoregions for 1997 to 2016. We scale the dry matter burned from the GFED4.1s inventory in each ecoregion by the ratios of predicted total burned areas under the natural climate scenario to those under the observed climate scenario, yielding the expected dry matter burned for the natural scenario.

### GEOS-Chem and Setup of Sensitivity Experiments.

We use the chemical transport model GEOS-Chem (version 14.1.1) to simulate the long-term smoke PM_2.5_ concentrations at the surface from 1997 to 2020. GEOS-Chem is a 3-D model of atmospheric composition driven by assimilated meteorological data from NASA Modern-Era Retrospective analysis for Research and Application version 2 (MERRA-2) (67). Here, we use the nested-grid configuration for North America domain (40.625° W to 140° W, 10° N to 69.5° N) under both observed and natural climate scenarios. This approach allows us to focus on the ACC impact on just fire emissions and not on smoke transport. For computational efficiency, we apply the aerosol-only version of GEOS-Chem, which simulates PM_2.5_ concentrations by considering the emissions of primary aerosol and aerosol precursors together with monthly mean oxidant fields archived from a GEOS-Chem benchmark simulation with a full chemical mechanism (68). The spatial resolution for the nested domain is 0.5° latitude × 0.625° longitude, with 47 vertical levels extended from the surface to 0.01 hPa. Initial and boundary conditions of chemical species concentrations are taken from global GEOS-Chem simulations at a resolution of 4° latitude × 5° longitude. The spin-up time for both global and nested-grid simulations is six months.

To attribute the contributions of ACC and NV on long-term smoke concentrations, we conduct three sensitivity experiments with different fire emission inventories. To isolate the impacts of fire emissions under the different climate scenarios, we use the same assimilated meteorological data to drive the sensitivity experiments. The control experiment (CTL) relies on the original GFED4.1s emissions derived from observed fire activity as the input for GEOS-Chem; this experiment attempts to best reproduce the smoke concentrations caused by observed wildfire conditions. This approach is supported by the good agreement between the burned areas from GFED4.1s inventory and the GPR-modeled burned areas using observed climate variables. For the natural experiment (NAT), we assume that the wildfires are under natural climate conditions, without being affected by ACC. The smoke concentration calculated in this experiment is due solely to natural wildfire emissions, constructed as described above. Fire emissions are emitted at the surface in the CTL and NAT experiments. Fire emissions are switched off in the background experiment (BKG). Sources of background PM_2.5_ in the western United States include dust, biogenic species, and anthropogenic emissions from the industrial, transportation, power plants, residential, and agricultural sectors. The main components of smoke PM_2.5_ are OA and BC, accounting for 80 to 90% of modeled smoke PM_2.5_ from 1997 to 2020. OA concentrations in the model are estimated from OC by applying a mass ratio of OA to OC of 2.1 (69). The differences between the CTL and BKG simulated PM_2.5_ concentrations represent smoke PM_2.5_ concentrations under the observed climate scenario, while differences between the NAT and BKG simulated PM_2.5_ concentrations represent smoke PM_2.5_ under the natural climate scenario. The contribution of ACC to smoke PM_2.5_ is estimated as the difference between the smoke PM_2.5_ concentrations in the observed and natural scenarios. To evaluate the CTL simulation, we compare the modeled OC and BC with surface measurements from the Interagency Monitoring of Protected Visual Environment (IMPROVE) program (*SI Appendix*, Fig. S20).

## Supplementary Material

Appendix 01 (PDF)

## Data Availability

The FPA-FOD burned area data are publicly available at https://www.fs.usda.gov/rds/archive/catalog/RDS-2013-0009.6 (48). The MTBS burned area data are publicly available at http://mtbs.gov/direct-download (70). The gridMET dataset is publicly available at https://www.climatologylab.org/gridmet.html (71). The CMIP6 data are publicly available at https://esgf-node.ipsl.upmc.fr/projects/cmip6-ipsl/ (72). NDVI data are publicly available at https://doi.org/10.7289/V5ZG6QH9 (60), https://doi.org/10.25921/gakh-st76 (61). The GFED4.1s fire emission inventory is publicly available at https://www.globalfiredata.org (73). The IMPROVE program data are publicly available at http://views.cira.colostate.edu/fed (74). The GEOS-Chem model outputs, burned area data predicted by GPR models, and estimated fire emissions under the natural climate scenario are publicly available at https://doi.org/10.7910/DVN/QPFDSI (75).
