# Supplementary material for: Large role of anthropogenic climate change in driving smoke concentrations across the western United States from 1992 to 2020
Source: Proc Natl Acad Sci U S A. 2025 Dec 2;122(49):e2421903122. doi: 10.1073/pnas.2421903122 (PMC12704757; doi:10.1073/pnas.2421903122)
Supplement: Supplementary file 1 — Appendix 01 (PDF) [file pnas.2421903122.sapp.pdf]

PNAS

Supporting Information for

Large role of anthropogenic climate change in driving smoke concentrations across the western United States from 1992 to 2020

Xu Feng[1,*], Loretta J. Mickley[1], Jed O. Kaplan[2], Makoto Kelp[3], Yang Li[4], Tianjia Liu[5]

[1]John A. Paulson School of Engineering and Applied Sciences, Harvard University, Cambridge, Massachusetts, USA
[2]Department of Earth, Energy, and Environment, University of Calgary, Calgary, Alberta, Canada
[3]Doerr School of Sustainability, Stanford University, Stanford, California, USA
[4]Department of Environmental Science, Baylor University, Waco, Texas, USA
[5]Department of Geography, University of British Columbia, Vancouver, British Columbia, Canada

*Xu Feng
Email: xfeng@g.harvard.edu

This PDF file includes:

Supporting text
Figures S1 to S20
Tables S1 to S3
SI References

Supporting Information Text

Evaluation of seasonality impacts on burned area predictions and anthropogenic climate change attribution

To evaluate the influence of seasonal variability in climate and vegetation variables on wildfire activity, we develop the Gaussian Process Regression (GPR) models using fire-season (May-October) data for climate, vegetation, and burned areas instead of annual data. Fig. S14 shows the predicted total burned areas compared to observations during the fire season from 1992 to 2020. We find that the GPR models can still capture interannual variability in fire-season burned areas with R^2 ranging from 0.35 to 0.89, which are comparable to the results predicted by GPR models with annual data (R^2 = 0.36-0.85). This consistency indicates that both annual-scale and seasonal-scale modeling approach proved sufficient for explaining interannual variability in burned areas at the ecoregion scale.

We also estimate the anthropogenic climate change (ACC) signals for climate variables and quantify ACC contributions to burned areas specifically during the fire season. Fig. S8 compares the spatial distributions of ACC signals for key climate drivers between the annual and fire-season averages. For T_{max} and VPD, the spatial patterns of ACC signals are broadly consistent between annual and fire-season averages, although the magnitudes of the fire-season ACC signals are notably greater, especially in the southern California and central regions. In contrast, the pattern and magnitude of ACC signals for accumulated precipitation differ significantly during the fire season. Anthropogenic climate forcing reduces the fire-season precipitation across northern California, Oregon, the Northern Rockies, and parts of the central region.

Despite these differences in the ACC signals in fire-season meteorology, we find that the overall ACC contribution to fire-season burned area in the western US remains stable, estimated at 65%, compared to 64% from the annual-scale modeling (Fig. S14). However, regional differences can be substantial. In the NW Forested Mts and Cold Deserts, the ACC contributions to fire-season burned areas increase substantially to 78% and 79%. In contrast, other ecoregions show reduced ACC contributions ranging from 20-36%, compared to 33-82% based on annual-scale estimates. Our results suggest that the estimates of the ACC attributions derived from annual mean meteorology may be conservative in the NW Forested Mts and Cold Deserts, where ACC-related trends are stronger, but could potentially overestimate attributions in the Mediterranean CA, SW Forested Mts and Central Prairies, where seasonal variability and fire-climate relationships differ. On the other hand, previous research has suggested that spring and wintertime meteorological

conditions play a strong role in grasslands such as the Central Prairies and at medium-high altitudes such as in the SW Forested Mts (1).

These regional differences in ACC attribution likely result from varying sensitivities of fire activity to fire-season climate conditions and ACC signals. To investigate these sensitivities, we use Partial Dependence Plots (PDPs) to assess the influence of individual predictors on predicted burned area from lightning- and human-ignited fires in both annual and fire-season GPR models (Fig. S15). To construct each PDP, we vary the value of one predictor across its observed range while holding all other predictors constant at their mean values, and we then input this modified dataset into the trained GPR model to obtain the corresponding predicted burned area. Use of the GPR model allows us to capture nonlinearities in predictor-response relationships. Fig. S15 also compares these predictor-response relationships between annual and fire-season models across the five ecoregions.

For lightning-ignited fires, the relationships between predictors and burned area are mostly linear across ecoregions, whereas human-ignited fires show stronger nonlinearities, perhaps reflecting the greater complexity of human interactions with environmental drivers. In the NW Forested Mts, lightning- and human-ignited fires show similar predictor-burned area relationships in both annual and fire-season models (Fig. S15a and S15b). The relationships between key predictors (e.g., VPD and T_{max}) and burned area are approximately linear; therefore, stronger ACC signals during the fire season yield larger ACC contributions to burned area, increasing from 62% to 78% (Fig. S15a). The Cold Deserts and Mediterranean CA show greater nonlinearity in predictor-burned area relationships (Fig. S15c-f), suggesting that such nonlinear relationships also shape seasonal differences in ACC attribution. In contrast, in the SW Forested Mts and Central Prairies, lightning-ignited fires do respond linearly to meteorological predictors, but the slopes in the fire-season models are shallower than in the annual models (Fig. S15g and S15i). For human-ignited fires in these two ecoregions, the burned area response is also less sensitive in the fire-season model to changes in most predictors (Fig. S15h and S15j). Consequently, even though ACC signals in VPD, T_{max}, and RH_{min} are stronger in the fire season, the corresponding changes in burned area are relatively smaller, leading to lower ACC contributions (28-36%). The differences in underlying predictor-burned area relationships between the annual and fire-season models account in part for the regional variability in ACC attribution.

Interpreting the nonlinearity in these predictor-response relationships can be challenging. The nonlinear response of burned area to some moisture variables such as relative humidity or precipitation is perhaps easiest to understand: high levels of moisture suppress fire activity by diminishing the availability of dry fuel, but very low levels of moisture can also

limit fire size by reducing vegetation cover (2). Detailed interpretation of these predictor-response relationships is beyond the scope of this paper.

Evaluation of vegetation moisture impacts on burned area predictions and ACC attribution

In addition to normalized difference vegetation index (NDVI), we test vegetation optical depth (VOD) as an input variable in the GPR models for both lightning- and human-ignited fires across the ecoregions. VOD is a proxy for total water content stored in the vegetation canopy, capturing vegetation moisture status. We use VOD data from the VOD Climate Archive (VODCA) v2 dataset, a multi-sensor, multi-frequency product derived from microwave Earth observations (3). This dataset provides daily VOD observations at 0.25° × 0.25° spatial resolution from 1987 to 2021. For this experiment, VOD is added to the original set of climate and vegetation predictors, including NDVI (Table S3). We then re-train the GPR models for each ecoregion.

Fig. S16 compares the annual total burned areas predicted by the new GPR models with VOD to the observations from the Fire Program Analysis-Fire Occurrence Database (FPA-FOD) from 1992 to 2020. We find that incorporating VOD improves model performance in the Ecoregion Mediterranean CA, where the R^2 increases to 0.53, suggesting a significant role for vegetation moisture in affecting the wildfire interannual variability. However, in other ecoregions, the inclusion of VOD has little effect on prediction of burned areas, with R^2 ranging from 0.43 to 0.82. This result implies that existing predictors such as vapor pressure deficit (VPD) and maximum air temperature (T_{max}) already serve as reasonable proxies for fuel moisture in those ecoregions.

We further evaluate the contributions of ACC to burned areas using the same attribution framework described in the Methods section. Fig. S16 shows that the estimated ACC contributions to burned areas remain broadly consistent with our original findings, ranging from 32% to 83% across ecoregions. As with the treatment of normalized difference vegetation index (NDVI), we assume that VOD is entirely attributable to the natural climate variability and apply the same VOD time series to both observed and natural climate scenarios in the GPR models. This assumption is necessary because VOD is not available from CMIP6 model outputs. As a result, ACC impacts on vegetation moisture are not explicitly separated in this analysis, and thus including VOD does not significantly alter the overall attribution of ACC in burned areas.

Comparison of burned area data from the FPA-FOD and the MTBS

We compare the annual total burned area over five ecoregions in western US from 1992 to 2020 based on the Fire Program Analysis-Fire Occurrence Database (FPA-FOD) and the Monitoring Trends in Burn Severity (MTBS) (Fig. S1). The temporal correlation coefficient between the two datasets is 0.98. The mean burned area from the FPA-FOD is 7% higher than that from the MTBS, which is likely explained by the lack of small fires (burned area < 1000 acres) in the MTBS dataset. Fig. S2A and S2B illustrate the consistent spatial distributions of accumulated burned areas of all fires in FPA-FOD and MTBS over 1992 to 2020, with a spatial correlation coefficient of 0.66.

Calculation of population-weighted smoke $PM_{2.5}$

We calculate the population-weighted smoke $PM_{2.5}$ concentrations using the CTL, NAT, and BKG simulations, combined with census data from the Gridded Population of the World version 4 (GPWv4) (4). The GPWv4 data, which have a spatial resolution of ~ 1 km × 1 km, are available for the years 2000, 2005, 2010, 2015, and 2020. We regrid the GPWv4 data to the spatial resolution of GEOS-Chem simulations (0.5° latitude × 0.625° longitude). The population-weighted smoke $PM_{2.5}$ concentration in each grid cell ($PPM_{2.5i}$) is calculated as follows:

$$PPM_{2.5i} = PM_{2.5i} \cdot (P_i / \sum_{i=1}^{N} P_i) \quad \text{(S1)}$$

$$PPM_{2.5} = \sum_{i=1}^{N} PPM_{2.5i} \quad \text{(S2)}$$

where $PM_{2.5i}$ is simulated annual mean smoke $PM_{2.5}$ concentration in the ith grid cell, P_i is the annual population in the ith grid cell, and $\sum_{i=1}^{N} P_i$ is the total population in a specific domain – e.g., the entire western US or the state of California – with N grid cells. The population-weighted smoke $PM_{2.5}$ concentration ($PPM_{2.5}$) in a specific domain is the sum of $PPM_{2.5i}$ over all grid cells within that domain (Eq. S2). In our study, the differences between the CTL and BKG simulated $PM_{2.5}$ concentrations represent smoke $PM_{2.5}$ concentrations under the observed climate scenario, while differences between the NAT and BKG simulated $PM_{2.5}$ concentrations represent smoke $PM_{2.5}$ under the natural climate scenario. The population-weighted smoke $PM_{2.5}$ concentration attributed to ACC is the difference in population-weighted smoke $PM_{2.5}$ concentration under the observed and natural climate scenarios.

Calculation of Vapor Pressure Deficit (VPD)

Using values from the sixth phase of the Coupled Model Intercomparison Project (CMIP6) simulations, we calculate VPD using monthly mean air temperature (T_{2m}) and relative humidity (RH_{2m}) at 2 m above ground as follows:

$$VPD = e_s(1 - RH_{2m}) \qquad (S3)$$

$$e_s = 611.2 \cdot \exp\left(17.67 \cdot \frac{T_{2m} - 273.15}{T_{2m} - 29.65}\right) \qquad (S4)$$

where e_s is the saturated vapor pressure.

Algorithm of GPR model

We apply GPR to model the relationships between burned areas (response variable) and multiple climate and vegetation variables (predictors). GPR is a non-parametric Bayesian model that defines a prior distribution over possible functions that fit the data, enabling flexible modeling of complex, nonlinear relationships without assuming a fixed functional form. The model assumes that the response variable y is generated from a latent function $f(x)$ with additive Gaussian noise:

$$y = f(x) + \varepsilon, \qquad \varepsilon \sim \mathcal{N}(0, \sigma_n^2) \qquad (S5)$$

The latent function $f(x)$ is modeled as a Gaussian Process, where any finite set of function values has a joint multivariate Gaussian distribution:

$$f(x) \sim \mathcal{GP}\big(m(x), k(x, x')\big) \qquad (S6)$$

Here $m(x)$ is the mean function and $k(x, x')$ is the covariance function that defines the similarity between any input vectors x and x'. In this study, we assume $m(x)$ to be zero and evaluate three commonly used covariance functions: squared exponential kernel (Eq. S7), exponential kernel (Eq. S8), and rational quadratic kernel (Eq. S9). For each GPR model, we select the covariance function that yields the best performance in reproducing burned area from the training data.

$$k(x, x') = \sigma_f^2 \exp\left(-\frac{\|x - x'\|^2}{2l^2}\right) \qquad (S7)$$

$$k(x, x') = \sigma_f^2 \exp\left(-\frac{\|x - x'\|}{l}\right) \qquad (S8)$$

$$k(x, x') = \sigma_f^2 \left(1 + \frac{\|x - x'\|^2}{2\alpha l^2}\right)^{-\alpha} \qquad (S9)$$

Here $\|x - x'\|$ is the Euclidean distance between input vectors; σ_f^2 is the signal variance; l is the characteristic length scale; and α is a scale-mixture parameter in the rational quadratic kernel that controls the relative contribution of large- and small -scale variations. These hyperparameters (σ_f^2, l, and α) are optimized during training by maximizing the marginal log-likelihood of the observed data. Table S3 shows the selected kernel functions for each ecoregion.

IMPROVE observations

The Interagency Monitoring of Protected Visual Environment (IMPROVE) network operates 122 sites in our study domain; most sites are distributed in wilderness and rural areas of western US. The IMPROVE sites collect 24-hour samples every three days. The quartz fiber filter in each sample is analyzed by thermal optical reflectance (TOR) for organic carbon (OC) and black carbon (BC). We exclude the sites with less than 50% valid monthly measurements during 1997 to 2020. There are 93 sites in total used for evaluating the CTL simulation (Fig. S20).

Validation of simulated aerosol concentrations

Fig. S20 compares the IMPROVE observations of surface OC and BC to results from the CTL simulation during wildfire season (June to October) in western US from 1997 to 2020. The CTL simulation generally reproduces the spatial distributions of observed OC and BC concentrations, including the hotspots in northern California, western Oregon, and northern Washington. The spatial correlation coefficients between the simulated and observed surface concentrations over all sites are 0.6 for OC and 0.64 for BC. However, the CTL simulation overestimates the surface concentration in northern California, where megafires have occurred in recent years. Model biases in this region may be due to underestimates of the plume injection heights during severe wildfire events. In other areas with smaller fire emissions, our simulation sightly underestimates the concentrations of OC and BC, likely due to low biases in anthropogenic emissions. Overall, the mean simulated concentrations of OC and BC over all sites during the wildfire season are 1.1 ± 1.4 μg m^{-3} and 0.14 ± 0.1 μg m^{-3}, in relatively good agreement with observed OC (1.5 ± 0.7 μg m^{-3}) and BC (0.2 ± 0.1 μg m^{-3}).

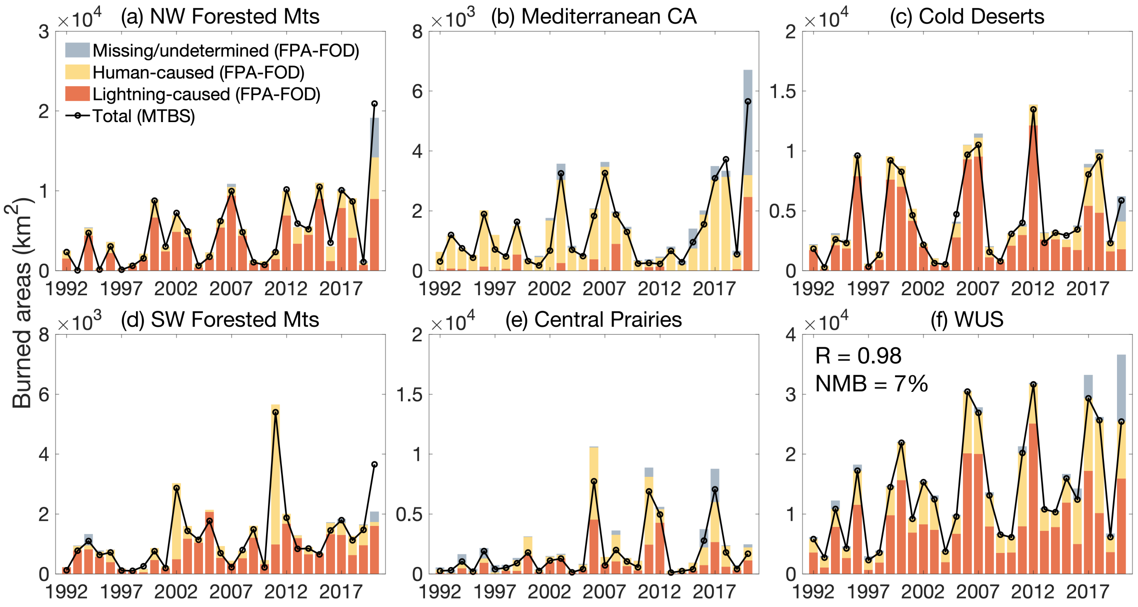

Fig. S1. Comparison of annual total burned areas from MTBS (black lines) and FPA-FOD (colored bars) in five ecoregions (a-e) and (f) the western US (the sum of five ecoregions) from 1992 to 2020. Values from the FPA-FOD dataset consist of the sum of burned areas from lightning-ignited fires (red), human-ignited fires (orange), and fires with missing and undetermined causes (grey). Range of values on y-axis varies among panels. Ecoregions are defined and shown in Figure 1A. The *R* and NMB shown inset compare the annual total burned areas in the western US in FPA-FOD to those in MTBS.

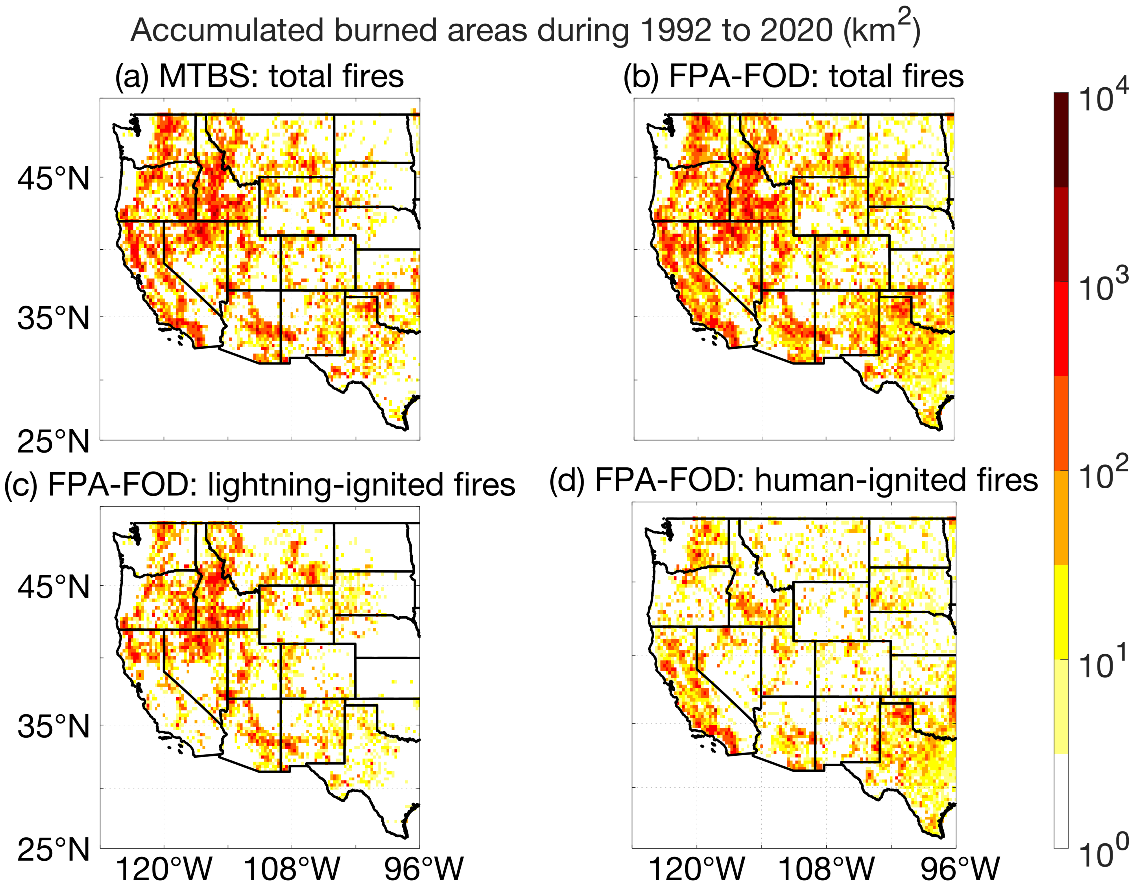

Fig. S2. Spatial distributions of burned areas of (a) total fires from the MTBS dataset, (b) total fires from the FPA-FOD dataset, (c) lightning-ignited fires from the FPA-FOD dataset, and (d) human-ignited fires from the FPA-FOD dataset, accumulated over 1992 to 2020.

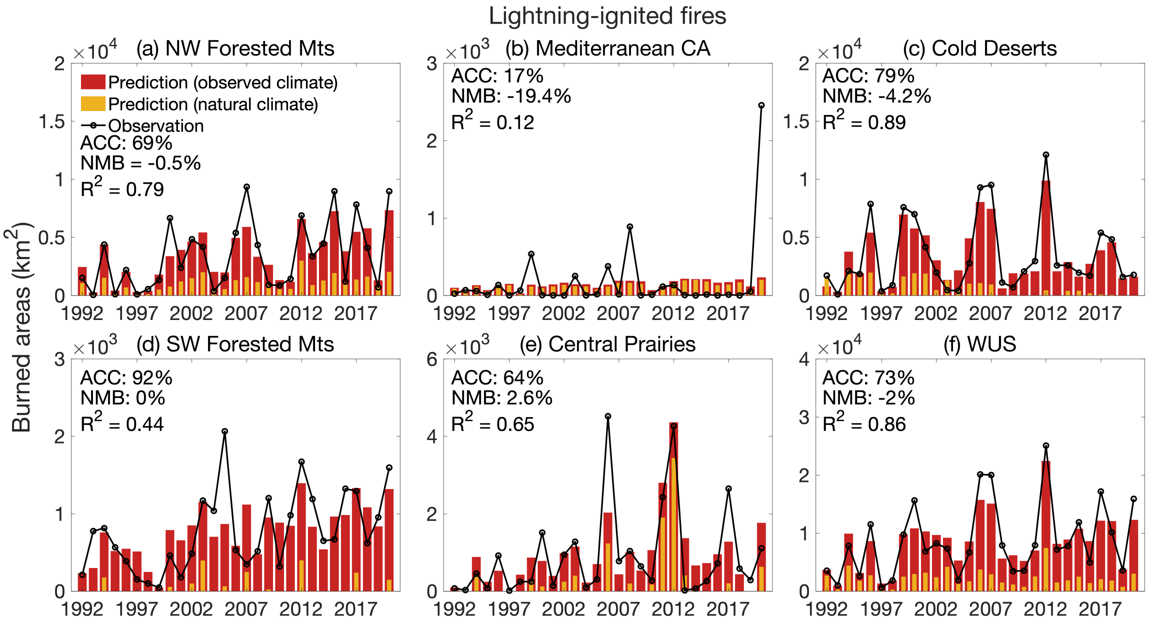

Fig. S3. Timeseries of annual total burned areas of lightning-ignited fires from 1992 to 2020 in five ecoregions in the western US (a-e), as well as over the West as a whole (f). Black curves represent observed burned areas, including both lightning-caused and human-caused fires, from the FPA-FOD dataset. Colored bars show the predicted timeseries of annual total burned area, calculated for observed (red) and “natural” (orange) conditions. The western US (WUS) represents the sum of burned area in the five ecoregions. The estimated percent contributions of ACC to the total predicted area burned are shown inset. The NMB and R^2 compare the predictions using observed climate conditions to the FPA-FOD dataset. Range of values on y-axis varies among panels. Ecoregions are defined in Figure 1A.

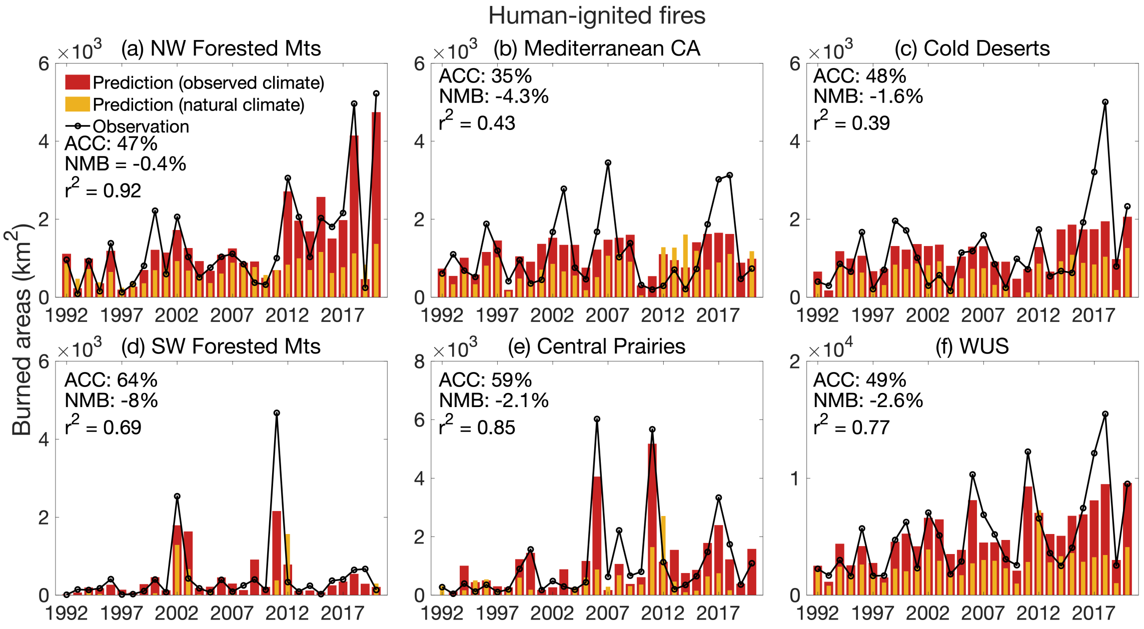

Fig. S4. Same as Fig. S3, but for annual burned areas of human-ignited fires.

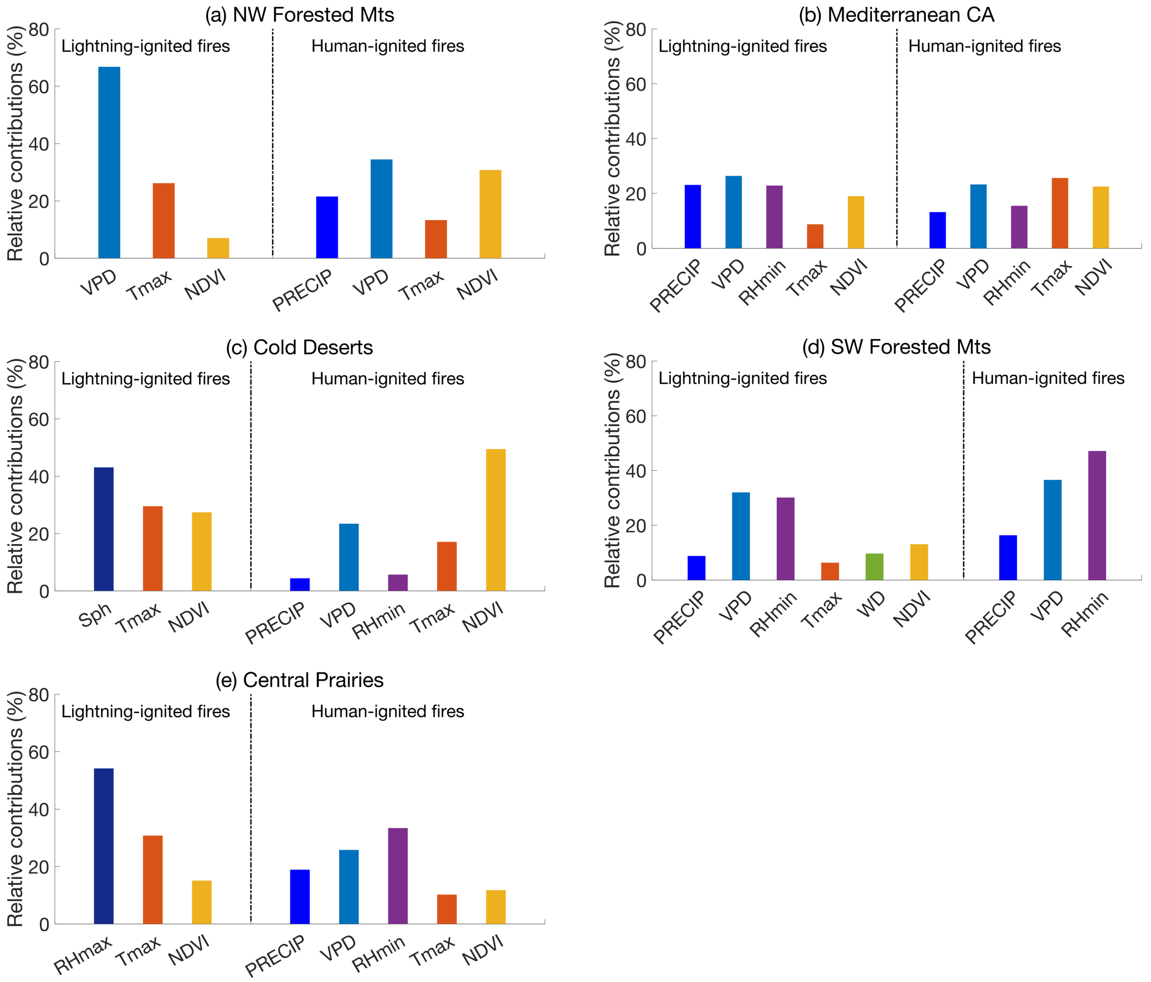

Fig. S5. Relative contributions of individual climate and vegetation predictors to burned areas predictions, derived using SHAP (Shapley Additive exPlanations) values from the trained GPR models. SHAP values are averaged from 1992 to 2020 and normalized to sum to 100% for each model. Results are shown separately for lightning-ignited fires (left panels) and human-ignited fires (right panels) in five ecoregions.

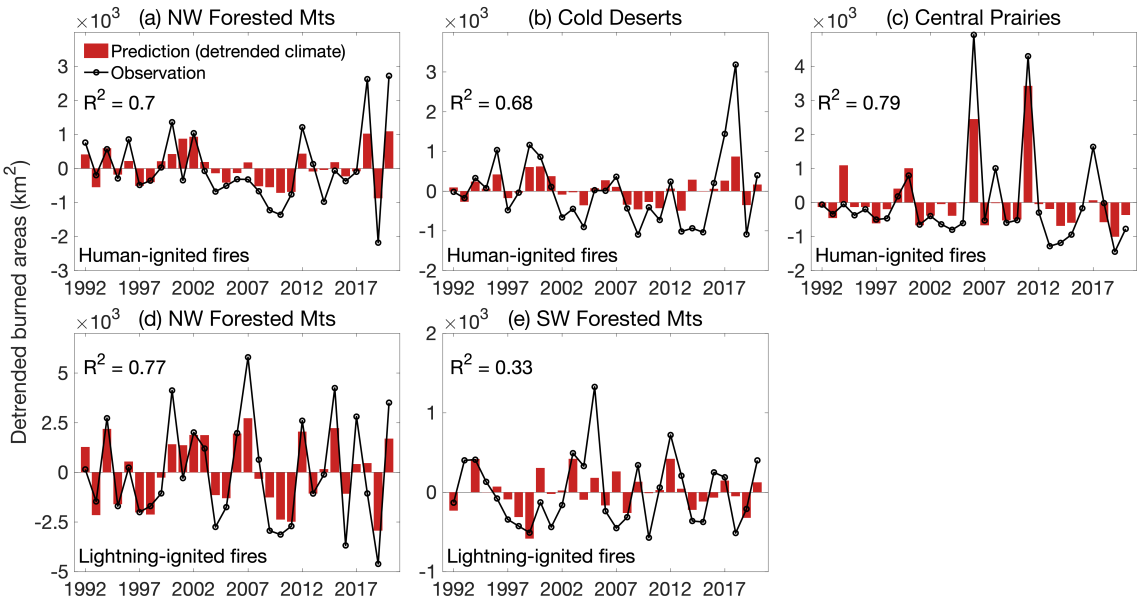

Fig. S6. Timeseries of detrended annual total burned areas for human-ignited and lightning-ignited fires in those ecoregions with significant long-term trends in burned areas from 1992 to 2020. Red bars represent departures from the long-term average of burned area calculated with detrended climate and vegetation data. Black lines represent departures from the long-term average of observed burned areas from the FPA-FOD dataset. The top panel shows results for human-ignited fires in the ecoregions (a) NW Forested Mts, (b) Cold Deserts, and (c) Central Prairies. The bottom panel shows results for lightning-ignited fires in the ecoregions (d) NW Forested Mts and (e) SW Forested Mts. The R^2 compares the predictions with the FPA-FOD observations.

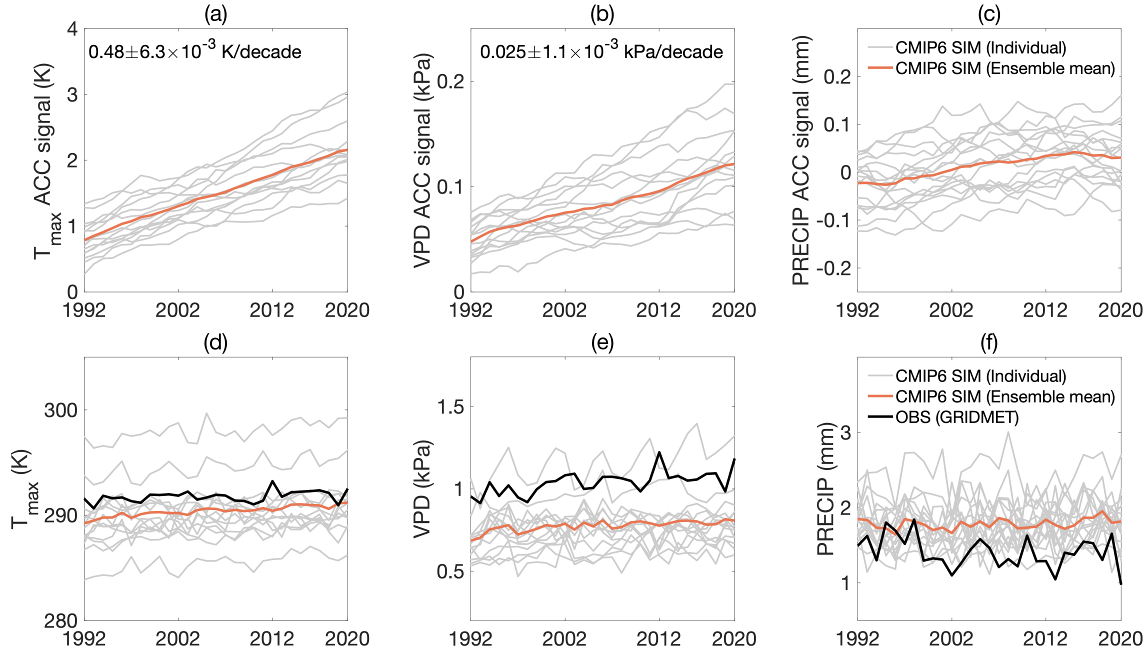

Fig. S7. The top panel shows timeseries of annual mean signals of ACC in (a) maximum air temperature (T_{max}), (b) vapor pressure deficit (VPD), and (c) daily accumulated precipitation (PRECIP), averaged over the western US from 1992 to 2020. We define the ACC signal as the absolute differences between subsequent 20-year moving averages of a specific meteorological variable and the mean value of that variable during the reference period (1921-1940). Each grey line represents the estimated ACC signal based on output from a single CMIP6 model. Red lines are the ensemble mean of all ACC signals for each year. The values inset represent the slopes and their uncertainties of the ensemble mean of ACC signals for T_{max} and VPD, which both show significant trends from 1992 to 2020. The slope is calculated using linear regression, and the uncertainty is determined at the 95% confidence interval. The mean trend in precipitation is not statistically significant. The bottom panel compares the timeseries of (d) T_{max}, (e) VPD, and (f) PRECIP from the CMIP6 simulations with observations from gridMET (black line) over the western US from 1992 to 2020. Each grey line represents the output from a single CMIP6 model. Red lines are the ensemble mean of all CMIP6 model outputs.

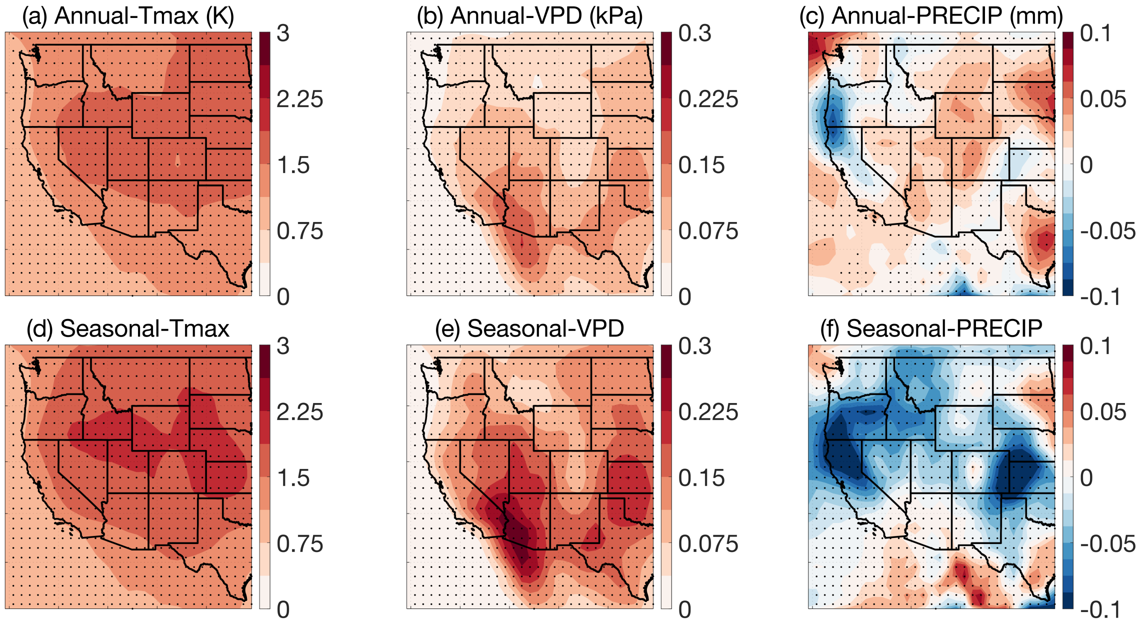

Fig. S8. Spatial distributions of ACC signals in the western US for (a, d) maximum air temperature (T_{max}), (b, e) vapor pressure deficit (VPD), and (c, f) accumulated daily precipitation (PRECIP), averaged from 1992 to 2020, derived from an ensemble of CMIP6 projections. The top and bottom panels show the ACC signal for (a-c) annual averages and (d-f) fire-season (May-October) averages. Black dots indicate grid cells where the trends in ACC signals are statistically significant, averaged across the ensemble of projections. Trends are calculated by linear regression with F-test ($p < 0.05$).

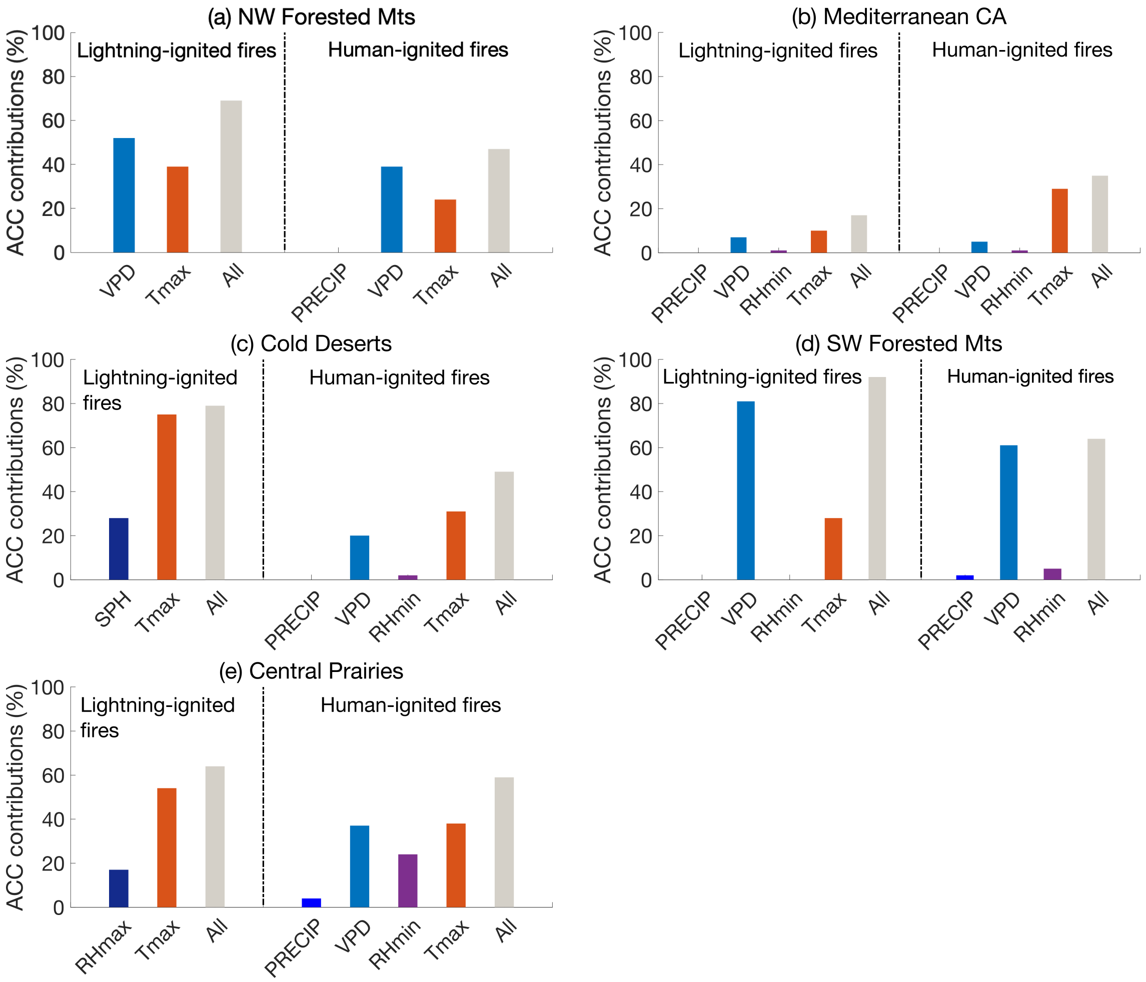

Fig. S9. The individual and combined ACC contributions of key meteorological variables to predicted burned areas of lightning-ignited and human-ignited fires in the five ecoregion regions from 1992 to 2020. To select the predictors in each region, we build Gaussian Processing Regression (GPR) models with different combinations of all variables and choose the one that yields the highest R^2 between the predicted and observed burned areas. T_{max} is daily maximum air temperature; VPD is daily mean vapor pressure deficit; RH_{max} and RH_{min} are daily maximum and minimum relative humidity, respectively; SPH is the daily mean specific humidity; and PRECIP is daily accumulated precipitation. Ecoregions are defined and shown in Figure 1A.

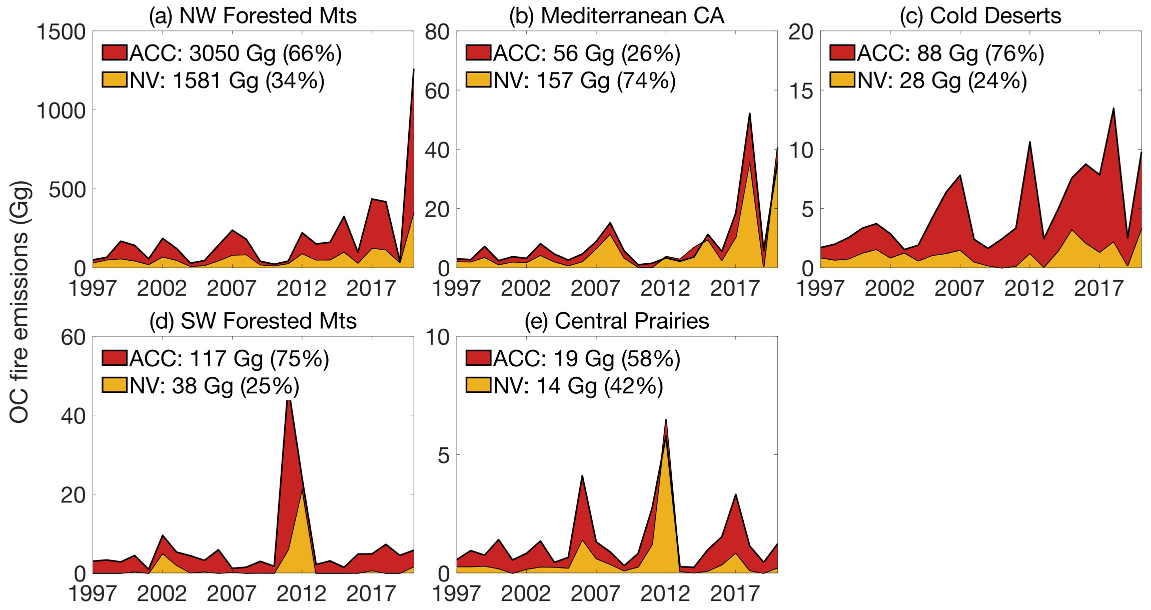

Fig. S10. Timeseries of annual total fire emissions of OC, the main component of smoke $PM_{2.5}$, under the observed climate conditions (sum of red and orange areas), as reported in the GFED4.1s inventory in five ecoregions from 1997 to 2020. Also shown are the OC fire emissions under the natural climate scenario (orange areas) and those emission attributed to ACC (red areas). Emissions for the natural scenario are derived using the GFEDF4.1s framework applied to the predicted natural area burned. The values inset represent the OC fire emissions in Gg and the percent contributions to total OC fire emissions due to ACC and to natural variability (NV). Ecoregions are defined and shown in Figure 1A.

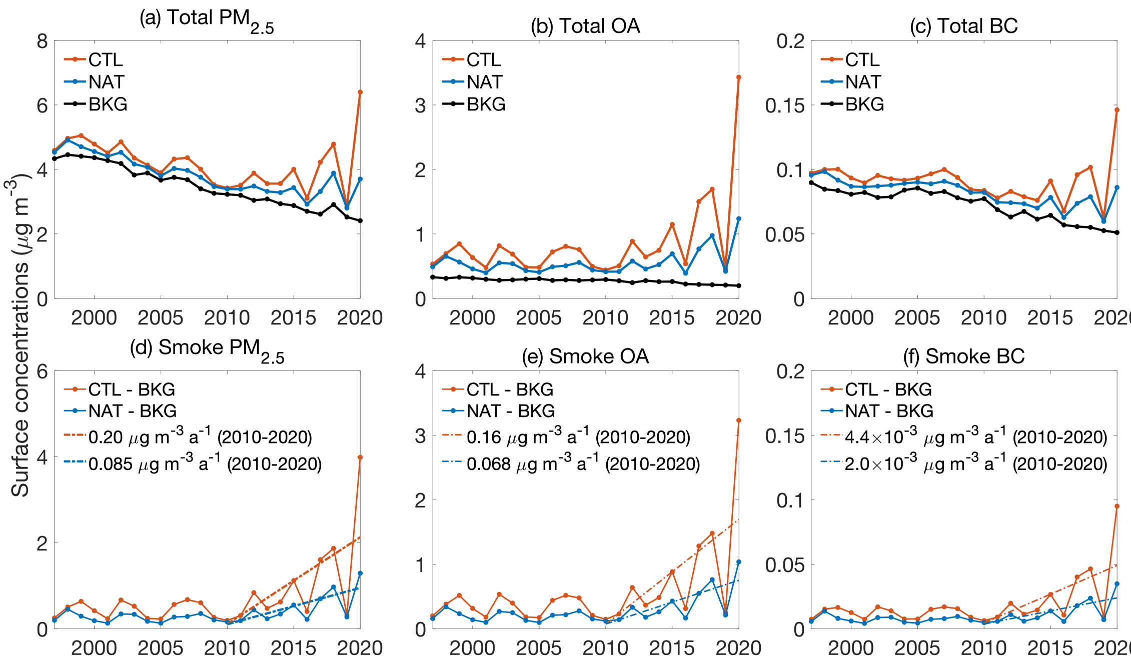

Fig. S11. Timeseries of simulated annual mean surface concentrations of (a) total $PM_{2.5}$, (b) total organic aerosol (OA), (c) total BC, (d) smoke $PM_{2.5}$, (e) smoke OA, and (f) smoke BC averaged across the western US from 1997 to 2020. Panels show results from three sensitivity simulations: control (CTL, red); natural (NAT, blue), which excludes the influence of ACC on wildfires; and background (BKG, black), which excludes all wildfire emissions. Annual mean concentrations of smoke $PM_{2.5}$, smoke OA, and smoke BC in panels (d), (e), and (f) are calculated as follows. The differences between the CTL and BKG simulations represent smoke $PM_{2.5}$, smoke OA, and smoke BC under the observed climate scenario, while differences between the NAT and BKG simulations represent smoke $PM_{2.5}$, smoke OA, and smoke BC under the natural climate scenario. Dashed lines in panels (d), (e), and (f) indicate the trends in annual mean smoke $PM_{2.5}$, smoke OA, and smoke BC from 2010 to 2020. The trends, shown inset, are calculated by Theil-Sen estimator and tested by the Mann-Kendall algorithm at a significant level of 0.05.

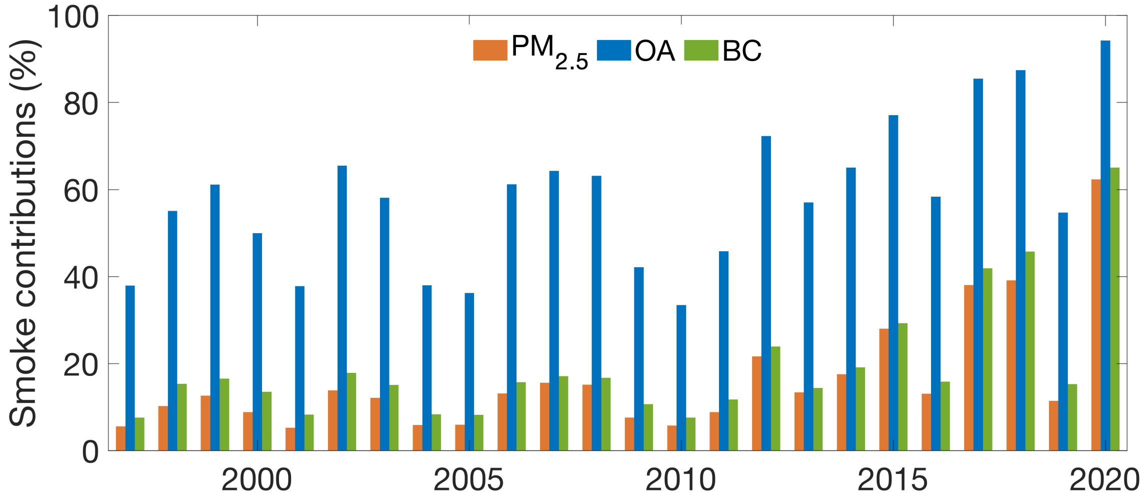

Fig. S12. Annual mean modeled contributions of smoke $PM_{2.5}$, smoke OA, and smoke BC to total annual mean values of these respective variables (smoke + non-smoke) under observed climate conditions in the western US from 1997 to 2020.

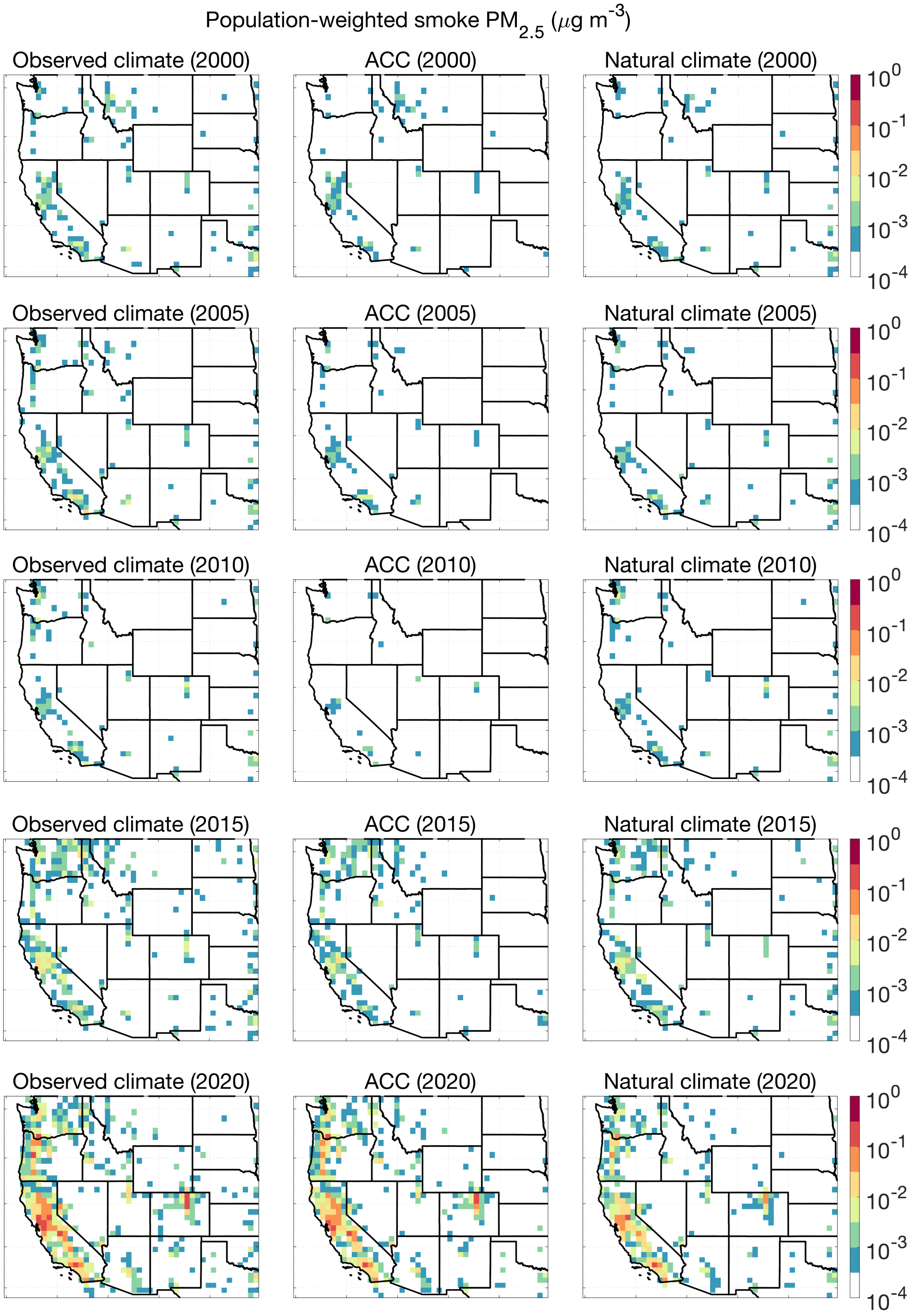

Fig. S13. Spatial distributions of the annual average contributions to population-weighted smoke concentrations over the entire western US in 2000, 2005, 2010, 2015, and 2020. The panels show the smoke concentrations weighted by population in each grid cell, with hot spots revealing where smoke concentrations affect the greatest numbers of people, compared to the rest of the West. The left and right columns show the population-weighted smoke $PM_{2.5}$ concentrations calculated by the CTL and NAT simulations. The center column shows the population-weighted smoke $PM_{2.5}$ concentrations attributed to ACC.

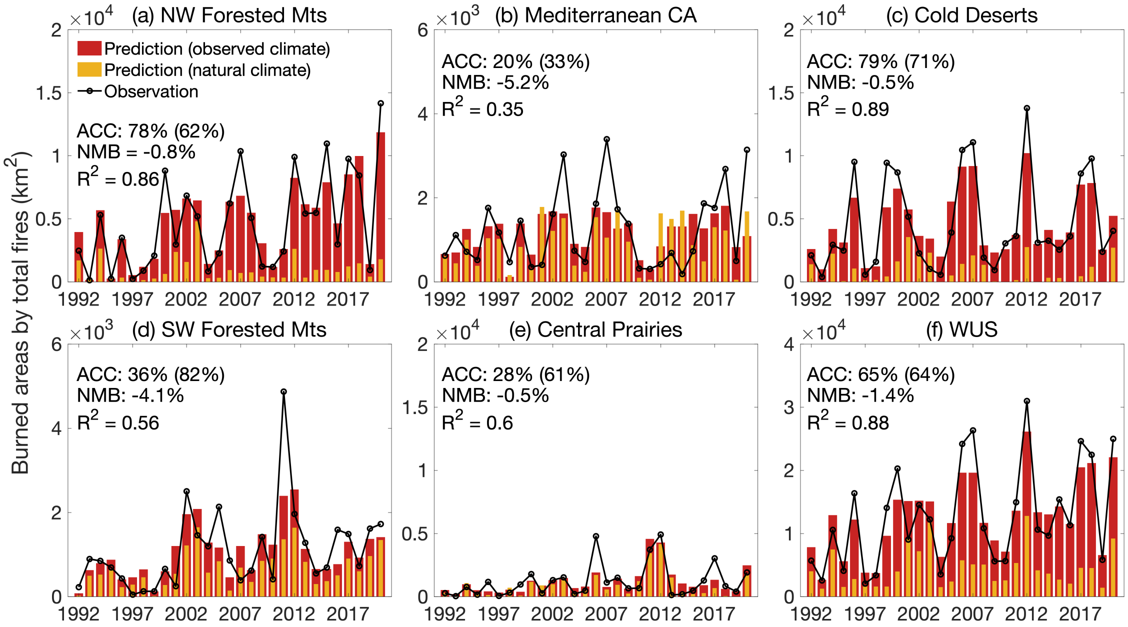

Fig. S14. Same as Figure 1B, but for total burned area from both lightning-ignited and human-ignited fires during the fire season (May to October) from 1992 to 2020. Percent values in parentheses are the contributions of ACC estimated by original GPR models using annual data.

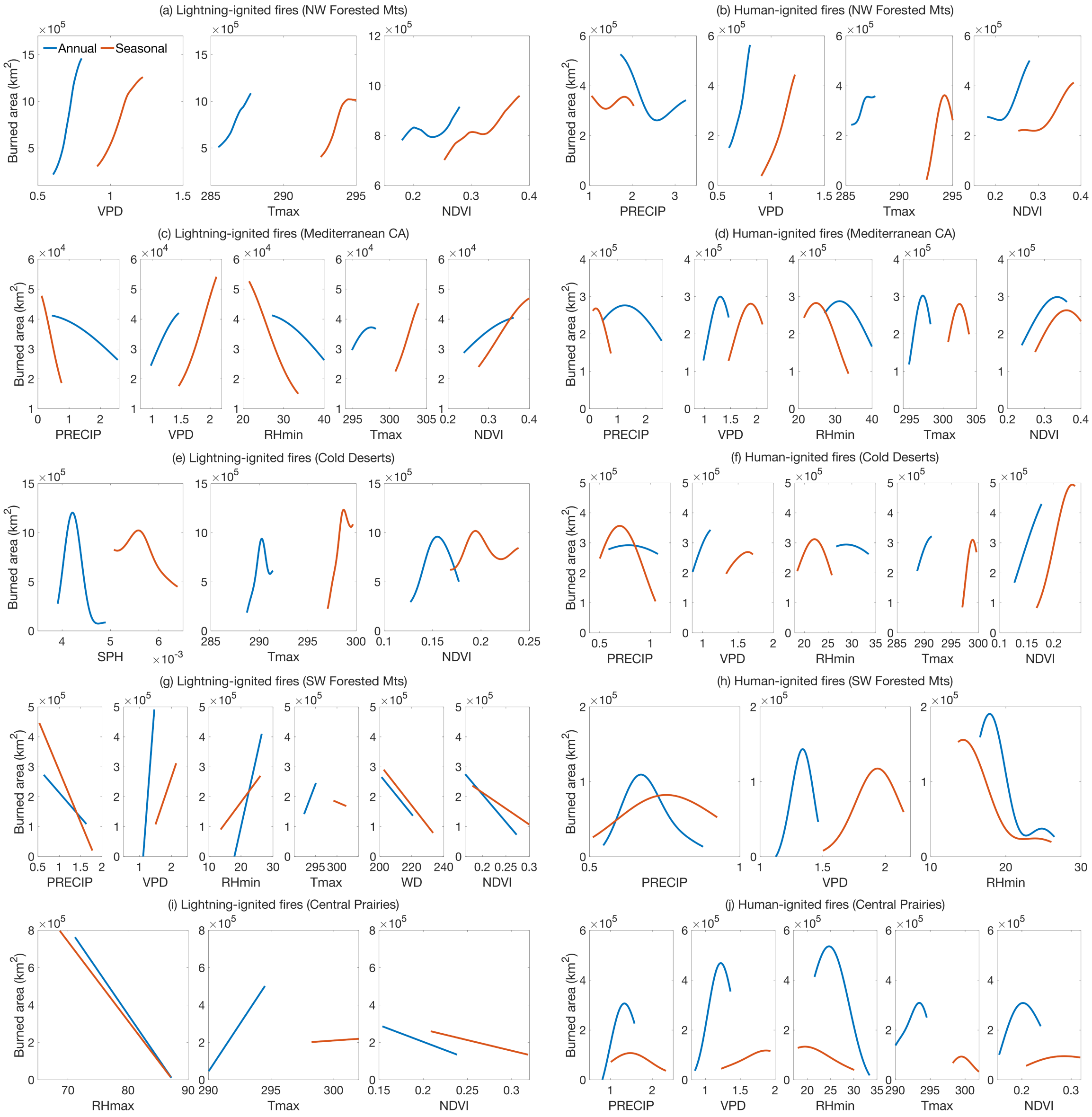

Fig. S15. Relationships between individual climate and vegetation predictors and predicted burned area derived from Partial Dependence Plots (PDPs). Results are shown for lightning-ignited fires (left column) and human-ignited fires (right column) across five ecoregions, based on original annual-scale GPR models (blue) and fire-season GPR models (red). PDP is constructed by varying a single predictor across its observed range while holding all other predictors at their mean values, and then applying the modified dataset to the trained GPR model to obtain the corresponding predicted burned area.

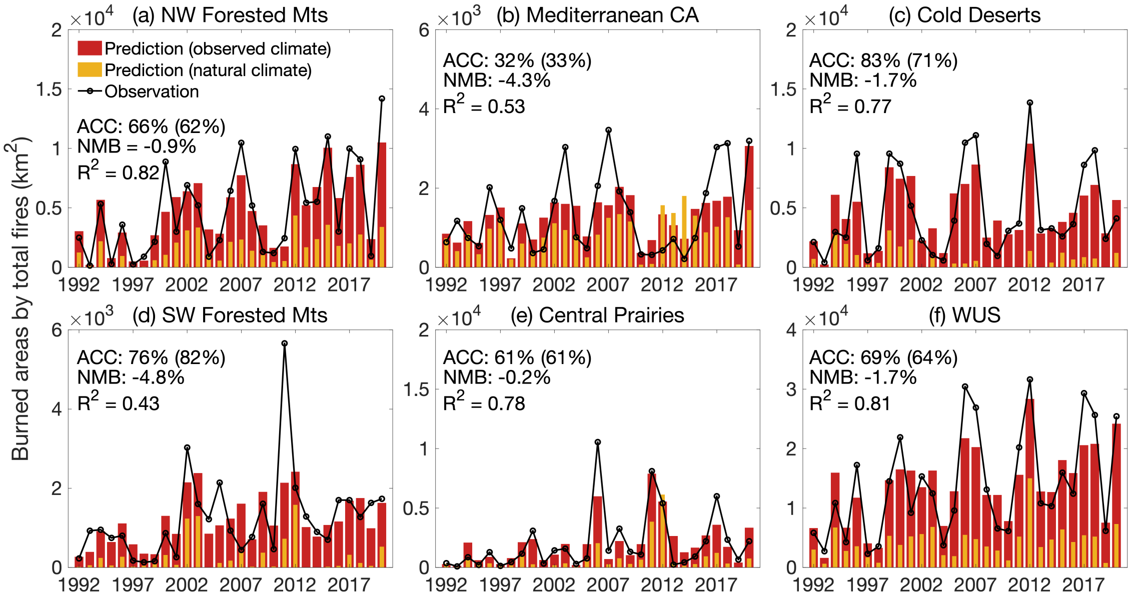

Fig. S16. Same as Figure 1B, but for annual total burned area from both lightning-ignited and human-ignited fires predicted by new GPR models incorporating vegetation optical depth (VOD). Percent values in parentheses are the contributions of ACC estimated by original GPR models without VOD.

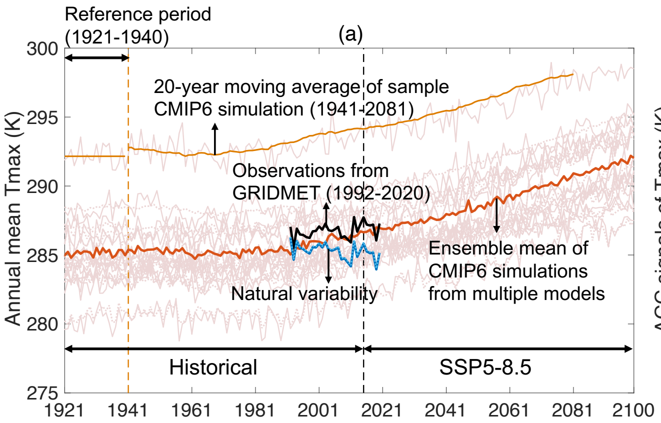

Fig. S17. Illustration of calculating the ACC signal in annual mean maximum air temperature (T_{max}) for the Ecoregion NW Forested Mts. (a) Timeseries of annual mean T_{max} from historical and SSP5-8.5 simulations (variant id: r1i1p1f1) from individual CMIP6 models (pink solid lines), the multimodel ensemble mean (red line), and observations from gridMET (black line). The pink dotted lines represent averages over all available realizations within each CMIP6 model. A 20-year moving average is applied to each individual model simulation to filter out the natural variability, as shown by the orange line in the sample CMIP6 simulation whose values represent outliers in the model ensemble. The residual between the observations and the ACC signal is defined as natural variability of T_{max} (blue line). The light blue dotted line represents the natural variability of T_{max} when the ACC signal is calculated from the realization-mean data (pink dotted lines). (b) ACC signal of T_{max} (purple line), defined as the multimodel ensemble mean of departures in 20-year moving averages from the reference period mean (1921-1940). Orange lines show ACC signals from individual CMIP6 models. The light purple dotted line represents the ACC signal calculated using realization-mean CMIP6 data.

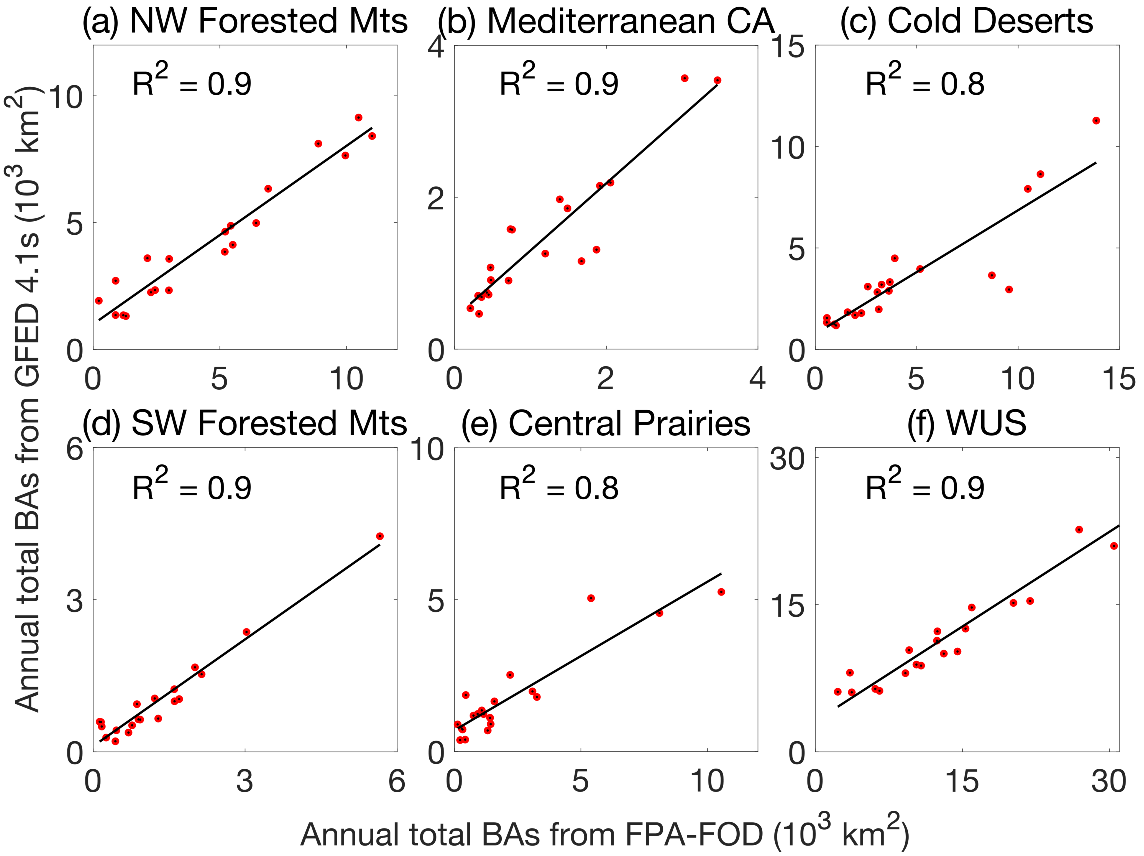

Fig. S18. Comparison of annual total burned areas (BA) from the GFED4.1s inventory versus those from the FPA-FOD dataset in five ecoregions (a-e) and (f) the western US during 1997 to 2016. Also shown are the R^2 between the two datasets and the fitted linear regression lines. Ecoregions are defined and shown in Figure 1A.

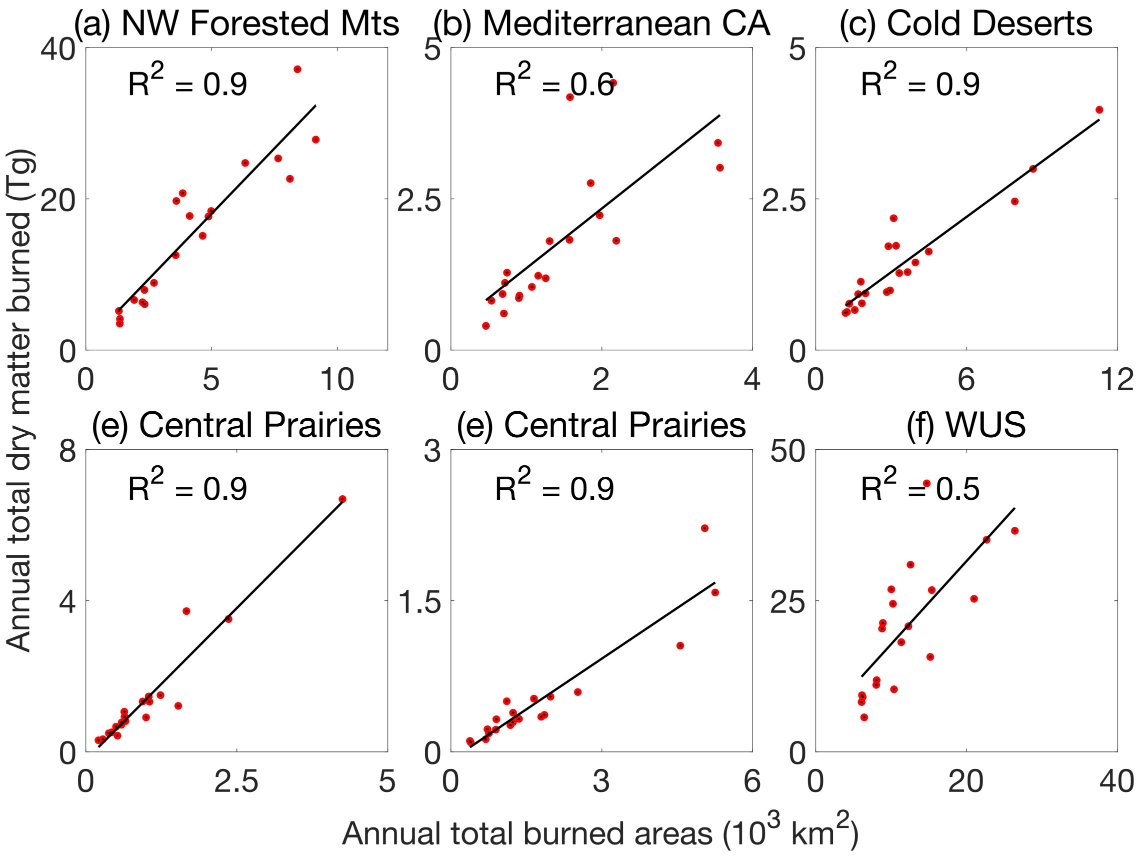

Fig. S19. Relationships between annual total burned areas and annual total dry matter burned in five Ecoregions (a-e) and (f) the western US during 1997 to 2016 from the GFED 4.1s inventory. Also shown are the R^2 and fitted linear regression lines. Ecoregions are defined and shown in Figure 1A.

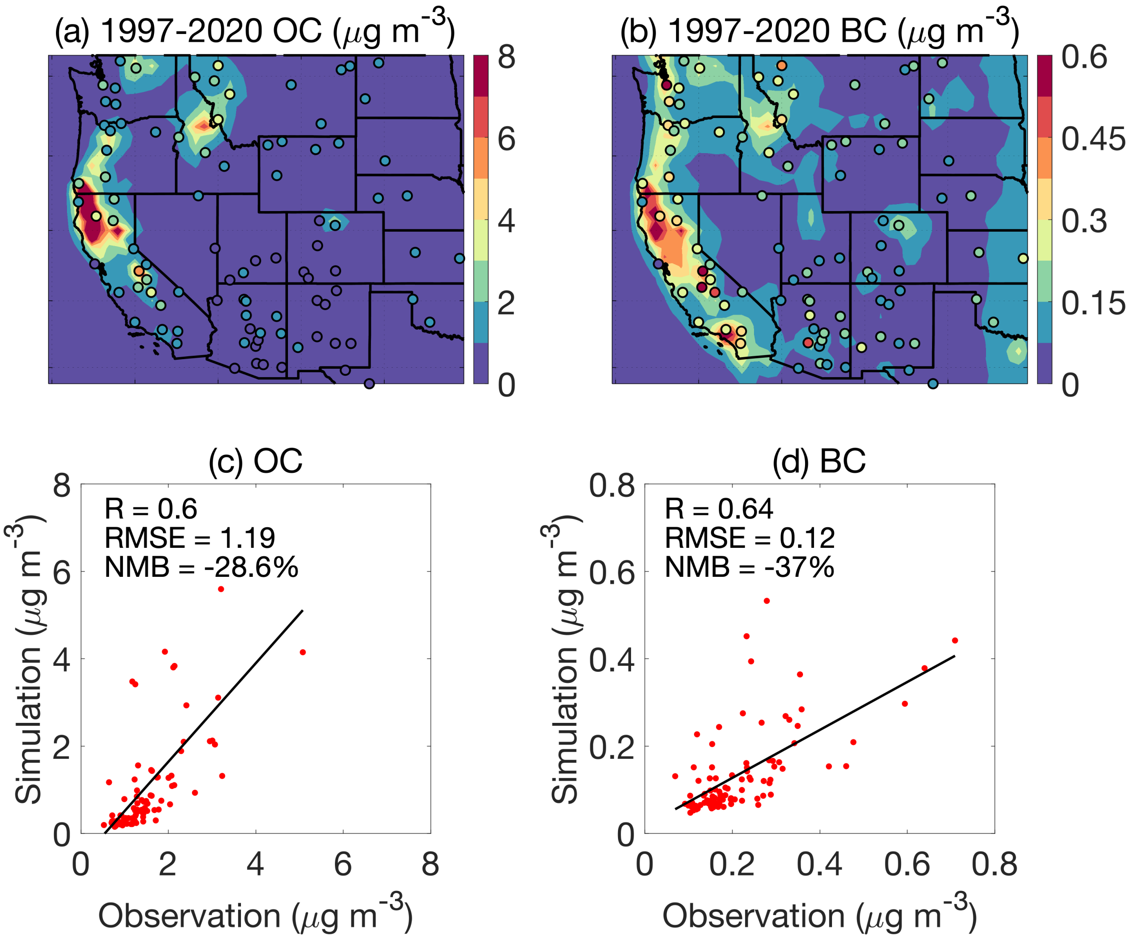

Fig. S20. Mean observed (symbols) and simulated (CTL experiment, filled contours) surface concentrations of (a) OC and (b) BC during the wildfire season (June to October) from 1997 to 2020. Scatter plots of observed and simulated mean concentrations of (c) OC and (d) BC at 93 sites in western US. The solid lines indicate the linear regression lines. Shown inset are the correlation coefficients, root mean square errors (RMSEs), and normalized mean biases (NMBs) of the simulated values compared to observations.

Table S1. Configurations of sensitivity experiments in this study.

Experiment	CTL	NAT	BKG
Time period	January 1997 to December 2020		
Spatial resolution	0.5° latitude × 0.625° longitude, 47 vertical levels		
Fire emissions	Original emissions from the GFED4.1s[1] (5)	Fire emissions under the natural climate scenario	Switched off
Anthropogenic emissions	CEDS v2[2] (global)(6) + EPA NEI 2016v1[3] (regional USA) (7)		
Biogenic emissions	MEGAN v2.1[4] (8)		
Meteorology	MERRA-2[5] (9)		
Chemical mechanism	GEOS-Chem aerosol-only simulation (10)		
Chemical boundary conditions	GEOS-Chem global simulation (4° latitude × 5° longitude)		

[1] Global Fire Emission Database version 4.1 with small fires
[2] Community Emissions Data System inventory version 2
[3] Environmental Protection Agency National Emissions Inventory 2016 version 1
[4] Model of Emissions of Gases and Aerosol from Nature version 2.1
[5] Modern-Era Retrospective analysis for Research and Application version 2

Table S2. List of the CMIP6 models used in this study. "X" indicates that the model provides the monthly mean output for the given variable in both the historical simulation (1920-2014) and the SSP5-8.5 simulation (2015-2100). Numbers in parentheses indicate the available realizations of tasmax for each CMIP6 model.

CMIP6 model name	Variables						
	tasmax[1]	tas[2]	hursmax[3]	hursmin[4]	hurs[5]	huss[6]	pr[7]
ACCESS-CM2	X (5)	X	X	X	X	X	X
ACCESS-ESM1-5	X (40)	X	X	X	X	X	X
AWI-CM-1-1-MR	X (1)					X	X
BCC-CSM2-MR	X (1)					X	X
CanESM5	X (50)	X	X	X	X	X	
CanESM5-1						X	
CAMS-CSM1-0							X
CAS-ESM2-0	X (2)	X			X	X	X
CESM2-WACCM		X			X	X	X
CMCC-CM2-SR5		X			X	X	X
CMCC-ESM2	X (1)	X	X	X	X	X	X
FGOALS-g3	X (4)	X	X	X	X	X	X
FIO-ESM-2-0	X (3)	X			X		X
IITM-ESM		X			X	X	X
MIROC6	X (50)	X	X	X	X	X	X
MPI-ESM1-2-HR	X (2)	X	X	X	X	X	X
MPI-ESM1-2-LR	X (30)	X	X	X	X	X	X
MRI-ESM2-0	X (5)	X	X	X	X	X	X
NESM3	X (2)						
NorESM2-LM						X	
NorESM2-MM						X	
TaiESM1						X	X

[1] Daily maximum near-surface air temperature (K)

[2] Near-surface air temperature (K)

[3] Daily maximum near-surface relative humidity (%)

[4] Daily minimum near-surface relative humidity (%)

[5] Near-surface relative humidity (%)

[6] Near-surface specific humidity (dimensionless fraction)

[7] Precipitation flux (kg m^{-2} s^{-1})

Table S3. Predictors and kernel functions for burned areas of lightning-ignited and human-ignited fires in the five ecoregions. Also shown are the R^2 and RMSE between the predictions from GPR models and the FPA-FOD observations, calculated with 5-fold cross-validation.

Ecoregion	Fire type	Predictors	Kernel	R^2	RMSE (km^2)
NW Forested Mts	Lightning	$\boldsymbol{T_{max}}$[1], VPD[2], NDVI[3]	Exponential	0.58	1.9×10^3
	Human	$\boldsymbol{T_{max}}$, VPD, PRECIP[4], NDVI	Rational quadratic	0.65	8.1×10^2
Mediterranean CA	Lightning	$\boldsymbol{T_{max}}$, VPD, $\boldsymbol{RH_{min}}$[5], PRECIP, NDVI	Rational quadratic	0.14	5.3×10^2
	Human	$\boldsymbol{T_{max}}$, VPD, $\boldsymbol{RH_{min}}$, PRECIP, NDVI	Squared exponential	0.17	8.5×10^2
Cold Deserts	Lightning	$\boldsymbol{T_{max}}$, SPH[6], NDVI	Squared exponential	0.28	2.7×10^3
	Human	$\boldsymbol{T_{max}}$, VPD, $\boldsymbol{RH_{min}}$, PRECIP, NDVI	Rational quadratic	0.23	8.9×10^2
SW Forested Mts	Lightning	$\boldsymbol{T_{max}}$, VPD, $\boldsymbol{RH_{min}}$, PRECIP, WD[7], NDVI	Squared exponential	0.34	4.0×10^2
	Human	VPD, $\boldsymbol{RH_{min}}$, PRECIP	Squared exponential	0.53	6.5×10^2
Central Prairies	Lightning	$\boldsymbol{T_{max}}$, $\boldsymbol{RH_{max}}$[8], NDVI	Exponential	0.61	7.3×10^2
	Human	$\boldsymbol{T_{max}}$, VPD, $\boldsymbol{RH_{min}}$, PRECIP, NDVI	Squared exponential	0.47	1.1×10^3

[1] Daily maximum near-surface air temperature

[2] Vaper pressure deficit

[3] Normalized difference vegetation index

[4] Daily accumulated precipitation

[5] Daily minimum near-surface relative humidity

[6] Specific humidity

[7] Wind direction

[8] Daily maximum near-surface relative humidity

SI References

1. R. Abolafia-Rosenzweig *et al.*, Winter and spring climate explains a large portion of interannual variability and trend in western U.S. summer fire burned area. *Environmental Research Letters* **17**, 054030 (2022).
2. A. Kuhn-Régnier *et al.*, The importance of antecedent vegetation and drought conditions as global drivers of burnt area. *Biogeosciences* **18**, 3861-3879 (2021).
3. R. M. Zotta *et al.*, VODCA v2: multi-sensor, multi-frequency vegetation optical depth data for long-term canopy dynamics and biomass monitoring. *Earth Syst. Sci. Data* **16**, 4573-4617 (2024).
4. Center for International Earth Science Information Network - CIESIN - Columbia University, Gridded Population of the World, Version 4 (GPWv4): Population Count, Revision 11. NASA Socioeconomic Data and Applications Center (SEDAC). https://doi.org/10.7927/H4JW8BX5. Deposited 20240730.
5. G. R. van der Werf *et al.*, Global fire emissions estimates during 1997–2016. *Earth Syst. Sci. Data* **9**, 697-720 (2017).
6. R. M. Hoesly *et al.*, Historical (1750–2014) anthropogenic emissions of reactive gases and aerosols from the Community Emissions Data System (CEDS). *Geosci. Model Dev.* **11**, 369-408 (2018).
7. E. P. Agency (2019) National Emission Inventory 2016v1 Platform.
8. A. B. Guenther *et al.*, The Model of Emissions of Gases and Aerosols from Nature version 2.1 (MEGAN2.1): an extended and updated framework for modeling biogenic emissions. *Geosci. Model Dev.* **5**, 1471-1492 (2012).
9. R. Gelaro *et al.*, The Modern-Era Retrospective Analysis for Research and Applications, Version 2 (MERRA-2). *Journal of Climate* **30**, 5419-5454 (2017).
10. E. M. Leibensperger *et al.*, Climatic effects of 1950–2050 changes in US anthropogenic aerosols — Part 1: Aerosol trends and radiative forcing. *Atmos. Chem. Phys.* **12**, 3333-3348 (2012).
